# Supplementary figures and images for: Nicotinamide metabolism is essential for Hepatitis C Virus replication and the production of infectious Lipo-Viro-Particles
Source: PLoS Pathog. 2026 Apr 22;22(4):e1014165. doi: 10.1371/journal.ppat.1014165 (PMC13138740; doi:10.1371/journal.ppat.1014165)

# Supplementary figure 1

A

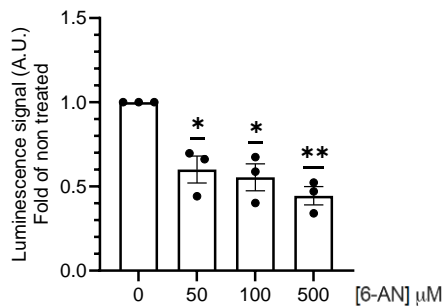

B

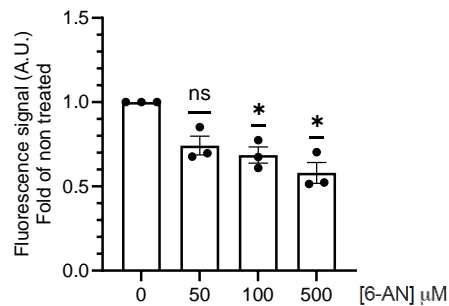

C

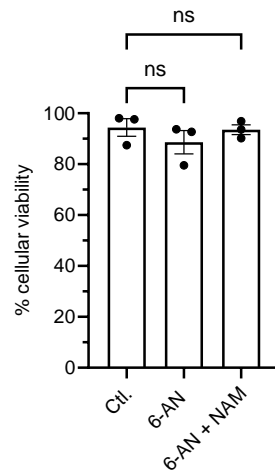

D

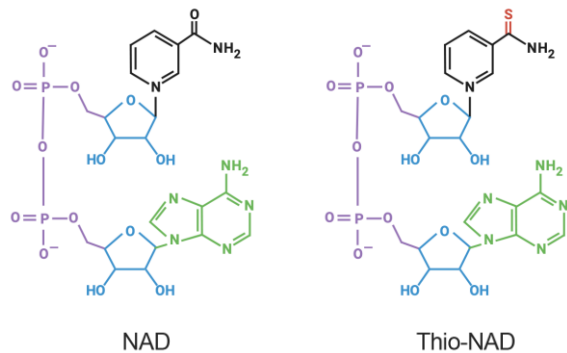

Supplement: S1 Fig — Intracellular ATP amounts were determined using CellTiter Glo assay (Promega) and cell proliferation after Hoechst staining of nuclei and quantification of fluorescence. Are presented means ± SEM (n = 3, one sample t-test, Bonferroni-Sidák adjusted p-value for multiple comparison to condition control, n.s. non significative, *p < 0.01). (C) Huh7 cells were cultured for 72 h in presence or not of 100 µM 6-AN ± 500 µM NAM, before cellular viability determination using CellTox Green Cytotoxicity Assay (Promega). Are presented means ± SEM (n = 3, one-way ANOVA for multiple comparison, n.s. non significative). (D) Molecular structures of NAD and Thio-NAD. Ribose in blue, adenine in green, pyrophosphate in purple, nicotinamide in black and additional thiol of thio-NAD in red. (PDF) [file ppat.1014165.s001.pdf]

# Supplementary Figure 2

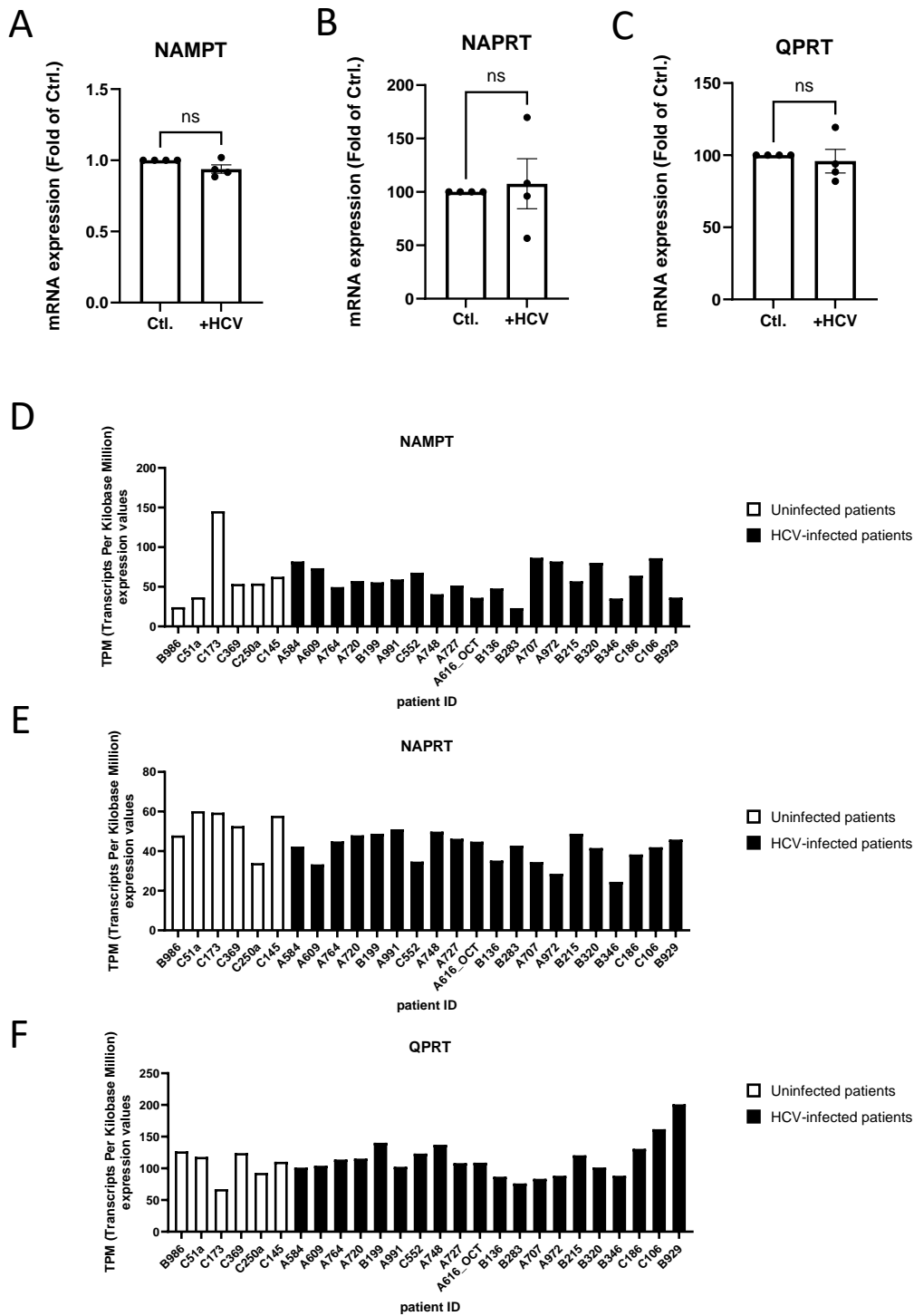

Supplement: S2 Fig — Seventy-two hours post-infection, total mRNA was extracted and NAMPT (A), NAPRT (B) and QPRT (C) expression was determined by RT-qPCR using RPL13A as housekeeping gene. Gene inductions are presented as fold of control. Are presented means ± SEM of three independent experiments. Student t test, non-significative (ns). (D-F) Expression levels of NAMPT, NAPRT and QPRT in liver biopsies of control and HCV infected patients (Data extracted from Boldanova T et al. study ([32]; GSE84346)). (PDF) [file ppat.1014165.s002.pdf]

# Supplementary Figure 3

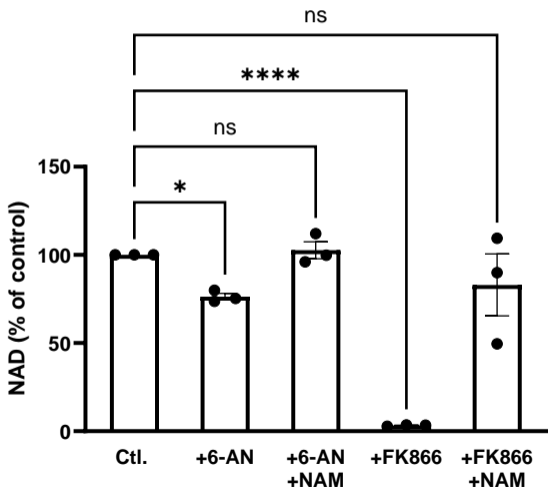

Supplement: S3 Fig — After treatment, the culture medium was removed and the cells were washed with PBS. Total NAD ⁺ /NADH was measured using the high sensitivity NAD/NADH Assay (Promega). For each treatment of cells, luminescence signal was measured and expressed as percentage of the untreated control. Data are presented as the mean ± SEM of three independent experiments and were analyzed using one way ANOVA, *p < 0.05, ****p < 0.0001, ns none significative. (PDF) [file ppat.1014165.s003.pdf]

Supplementary Figure 4

A

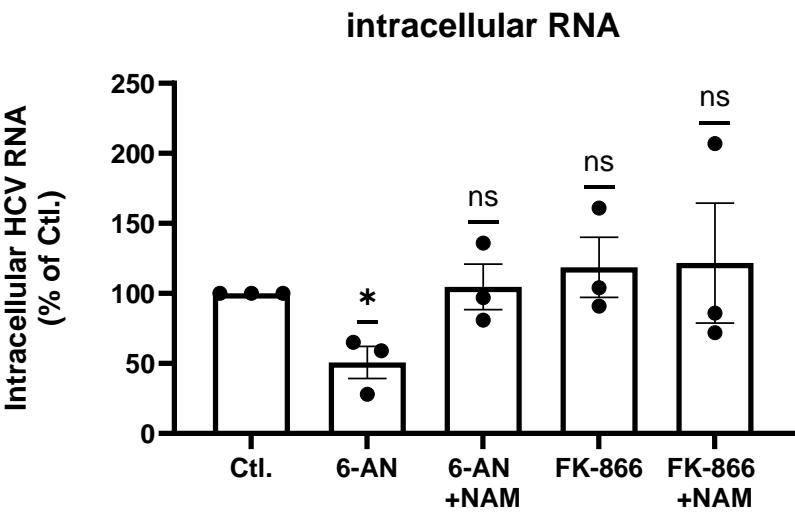

B

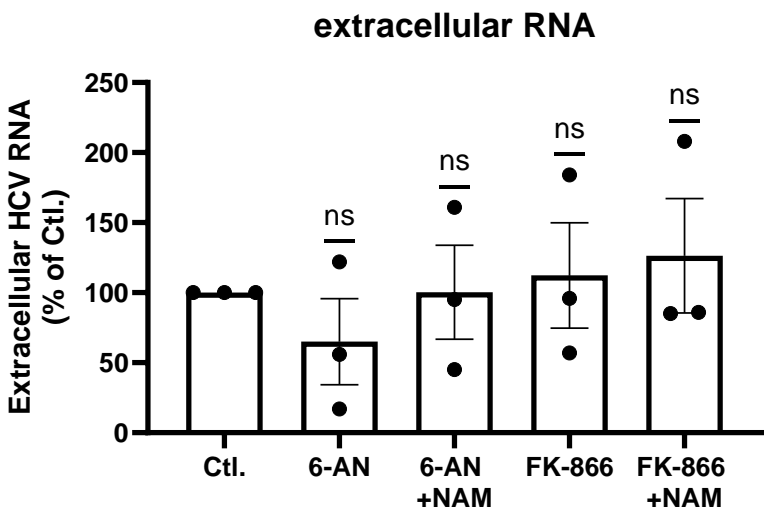

C

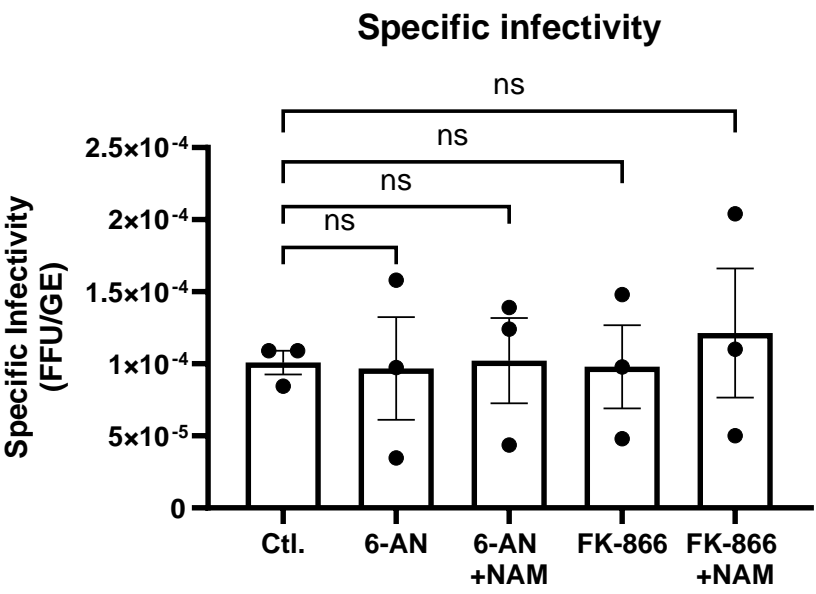

Supplement: S4 Fig — Seventy-two hours post infection, culture medium was replaced and 6-AN (100 μM) or FK-866 (100 nM), supplemented or not with NAM (500 μM), were added to the cultures. After 48 h of treatment, intracellular (A) and extracellular (B) viral RNA were quantified by qPCR and specific infectivity of viral particles in supernatants was determined (C). Are presented means ± SEM (n = 3). A and B, one sample t-test, *p < 0.05, ns none significative. C, One-Way Anova with Sidak correction for multiple comparisons, ns none significative. (PDF) [file ppat.1014165.s004.pdf]

Supplementary Figure 5

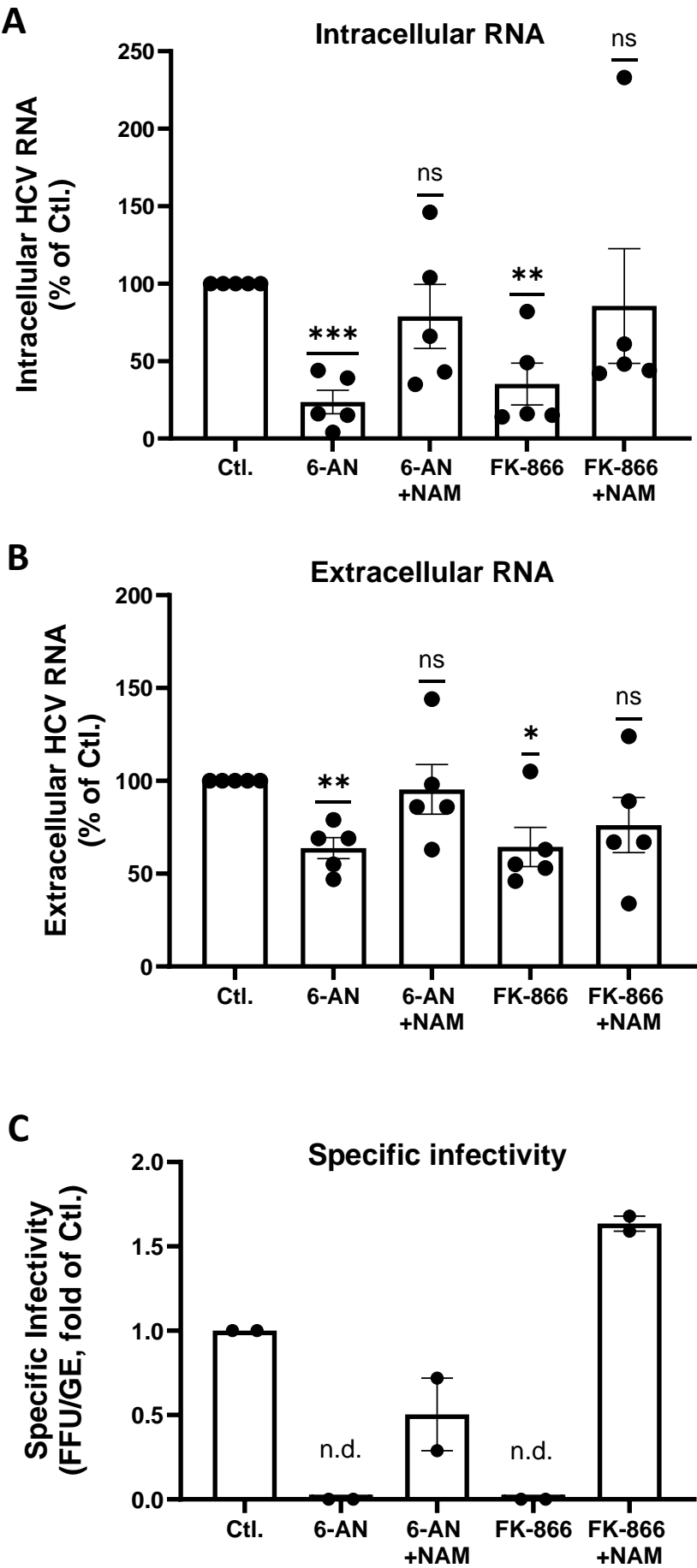

Supplement: S5 Fig — The cells were then treated for 72 hours with either 100 µM 6-AN or 100 µM 6-AN + 500 µM NAM. (A) Intracellular and (B) extracellular HCV-RNA were quantified by qPCR (n = 5). (C) Viral titer was determined on Huh7.5 cells and specific infectivity calculated as the ratio of FFU/ HCV genomes within supernatant (n = 2). Are presented means ± SEM, n.d.: ratio none determined due to FFU = 0. (PDF) [file ppat.1014165.s005.pdf]

# Supplementary Figure 6

A

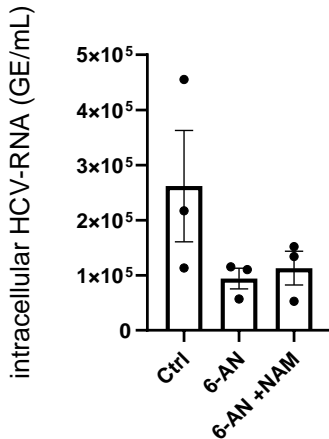

B

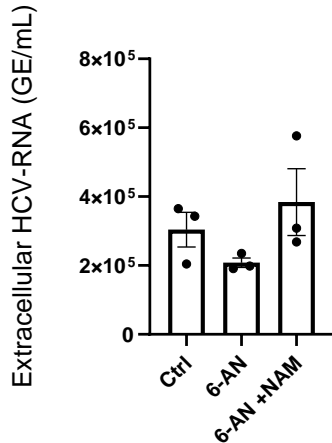

Supplement: S6 Fig — Twenty-four hours post-infection molecules or the solvent alone (Ctrl) were added at the final concentration of 100μM 6-AN ± 500μM NAM. Three days post infection, cells (A) and culture supernatants (B) were harvested for RNA extraction and HCV genomes were quantified by qPCR. Are presented means ± SEM of 3 biological replicates proceeded in the same experiment. (PDF) [file ppat.1014165.s006.pdf]

# Supplementary Figure 7

A

Cell count

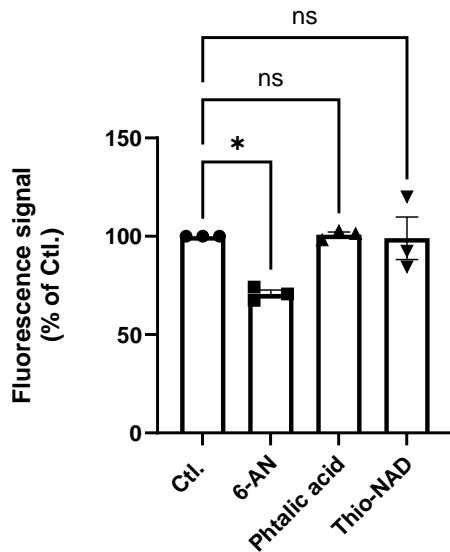

B

Intracellular ATP

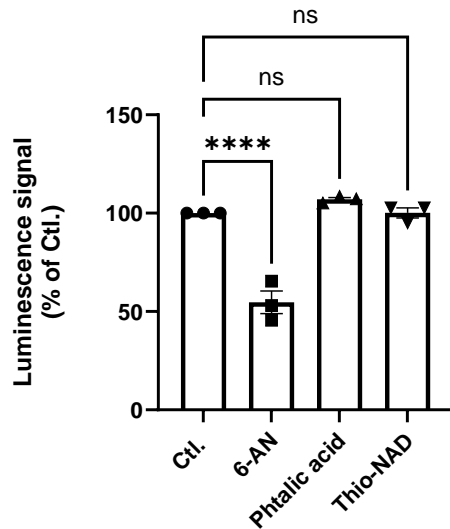

Supplement: S7 Fig — (A) Cell proliferation was determined after Hoechst staining of nuclei and quantification of fluorescence. (B) Intracellular ATP amounts were determined using CellTiter Glo bioluminescent assay. Are presented means ± SEM, n = 3, one-way ANOVA, Bonferroni adjusted p-value for multiple comparison. ns non significative, *p < 0.05, ****p < 0.0001. (PDF) [file ppat.1014165.s007.pdf]

A

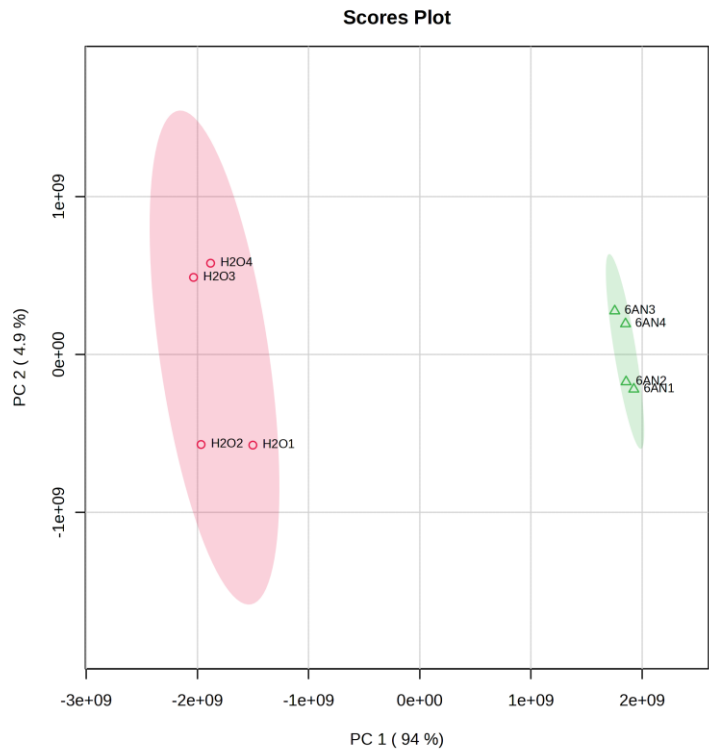

B

Metabolites sets

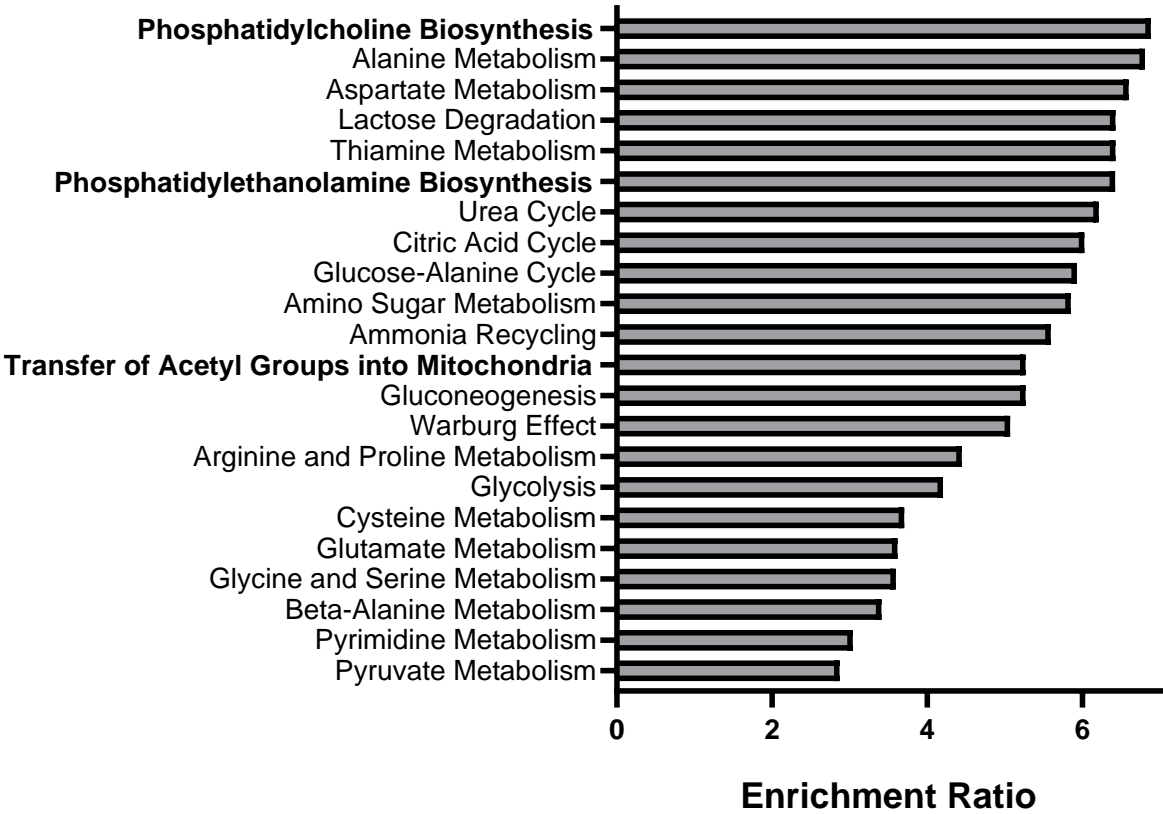

Supplement: S8 Fig — Are compared quantities of cellular metabolites determined for Huh7 cells treated for 72h with 100µM 6-AN and control cells. Red cycles correspond to control samples whereas green triangle correspond to 6-AN treated samples. (B) Differentially represented identified-metabolites with a p-value < 0.05 were submitted to Metaboanalyst server (6.0 release) for standard MSEA analysis. Enrichments ratios were computed by observed hits/ expected hits and the 22 pathways with a significative enrichment ratio (FDR < 0.05), were ranked and presented from the higher ratio (top of the list). In bold are pathways related to phospholipid metabolism. (PDF) [file ppat.1014165.s008.pdf]

# Supplementary figure 9

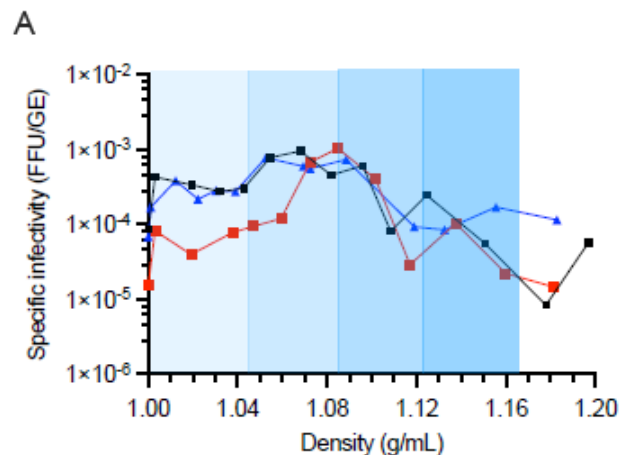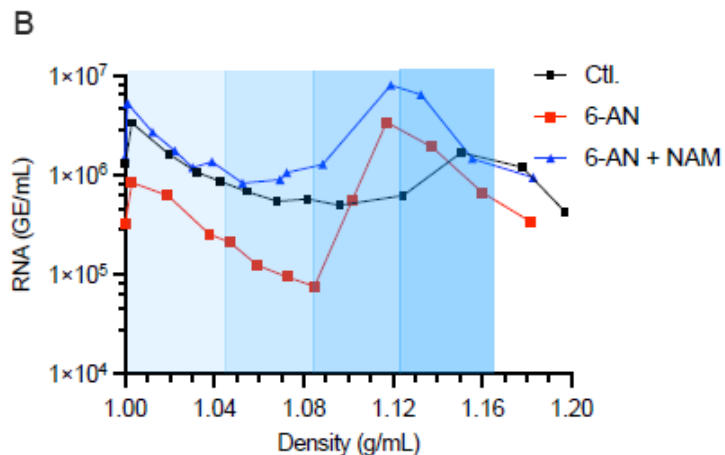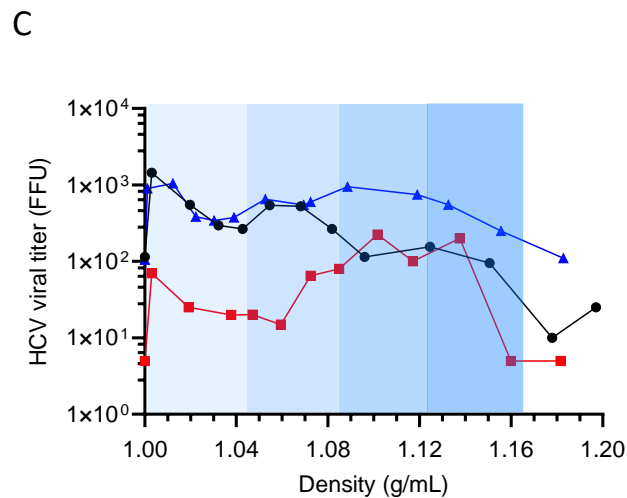

Supplement: S9 Fig — 1ml of ten-fold concentrated cell-culture supernatants were separated on iodixanol gradient (3–40%). Specific infectivity of viral particles (A), HCV RNA content (B) and FFU (C) were determined in each of the collected density fractions. (PDF) [file ppat.1014165.s009.pdf]
