# Supplementary material for: Nicotinamide metabolism is essential for Hepatitis C Virus replication and the production of infectious Lipo-Viro-Particles
Source: PLoS Pathog. 2026 Apr 22;22(4):e1014165. doi: 10.1371/journal.ppat.1014165 (PMC13138740; doi:10.1371/journal.ppat.1014165)
Supplement: Attachment — Submitted filename: Response to Reviewers 21-12-25.pdf [file ppat.1014165.s012.pdf]

PPATHOGENS-D-25-01682
Nicotinamide metabolism is essential for Hepatitis C Virus replication and the production of infectious Lipo-Viro-Particles
PLOS Pathogens

Dear Dr. Volker Lohmann,
Please find attached the revised version of our manuscript PPATHOGENS-D-25-01682, entitled "Nicotinamide metabolism is essential for Hepatitis C Virus replication and the production of infectious Lipo-Viro-Particles."
We have provided a detailed, point-by-point response to the reviewers' comments. All concerns raised by both reviewers have been addressed, and the additional key experiments requested have been performed. Notably, new data validating our findings in differentiated cells, as well as the analysis of viral factories by immunolabeling, have been incorporated into the revised manuscript.
All changes made to the manuscript are clearly indicated and detailed below. We sincerely thank the reviewers for their constructive and insightful comments, which have significantly improved the quality of our work.
We hope that the revised manuscript is now suitable for publication.
Yours sincerely,
Dr. Olivier Diaz

Reviewer #1:

1. The main issue is that HCV infection kinetics depend on cell growth and cell density. For example, when during RNAi experiments cells growth is slightly reduced (see, e.g., PMID: 25616068). Thus, all detrimental effects observed on HCV infection could be due to the effect the inhibitors have on cell growth. The authors need address this issue e.g., by using DMSO to arrest the cell cycle and then perform infection and treatment experiments as a control.

As requested, Huh7 cells were differentiated in DMSO and the effect of inhibiting nicotinamide metabolism on HCV infection was tested. First, Huh7 cells were cultured in the presence of 2% DMSO for 7 days, and were subsequently infected at a MOI of 0.5. One day after infection, cells were treated with either 6-AN or the NAMPT inhibitor, FK-866. Treatment was applied for 7 days before collecting cells and supernatants. Intracellular and secreted viral RNA were quantified by RT-qPCR. In addition, infectious particles in culture supernatants were titrated.

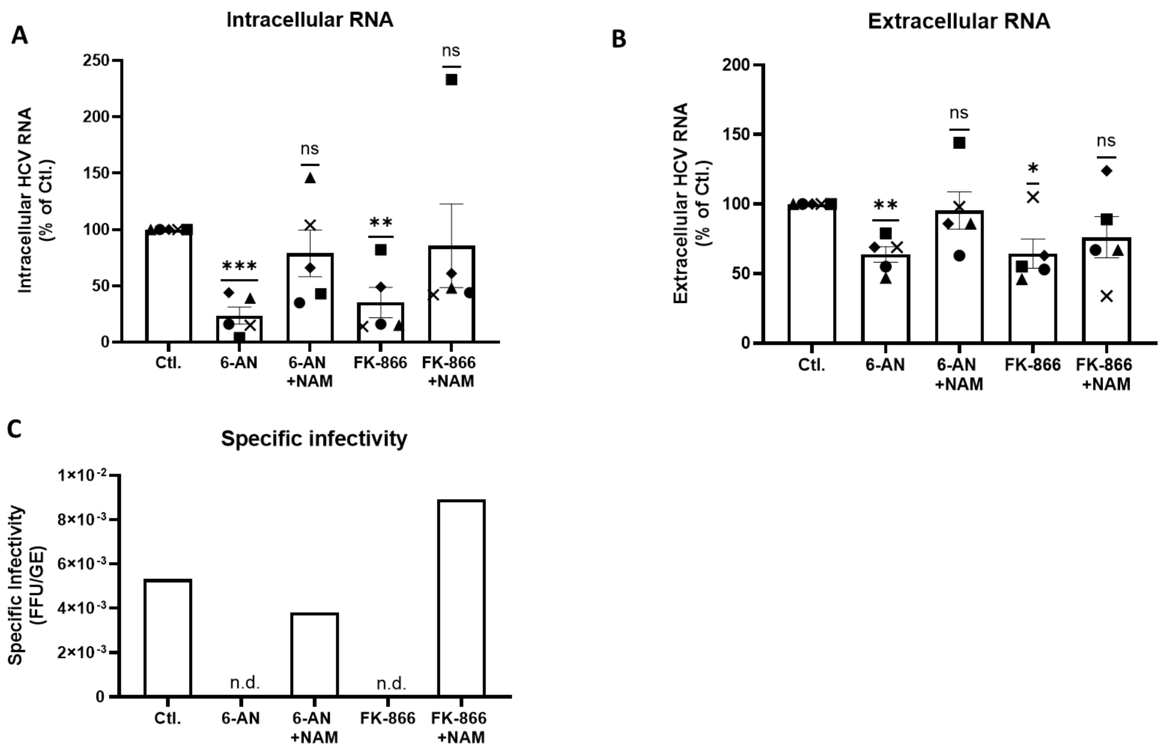

Figure R1: *Huh7 cells were seeded in a 24-wells plate and cultured for 7 days in complete medium with 2% DMSO. The cells were then infected and treated for 7 days with either 100 µM 6-AN or 100 µM 6-AN + 500 µM NAM. Medium was renewed at day 3 post-infection. (A) Intracellular and (B) extracellular HCV-RNA were quantified by qPCR (n=5). One sample t-test, ns, none significative, *p<0.05, **p<0.01, ***p<0.001. (C) Viral titer was determined on Huh7.5 cells and specific infectivity calculated as the ratio of FFU / HCV genome (GE) within supernatant (n=2). Are presented means ± SEM, n.d.: no infectious viral particle detected.*

Intracellular RNA levels decreased in cells treated with 6-AN or FK-866, whereas addition of NAM was able to restore replication as expected (Fig. R1A). The overall effect on the secretion of viral RNA was limited (Fig. R1B), but the specific infectivity of secreted virions was drastically reduced when nicotinamide metabolism was inhibited (Fig. R1C). This loss of viral infectivity is even more pronounced in differentiated cells: although viral RNA is still released into the culture medium following treatment with 6-AN or FK-866 (Fig. R1C), the number of secreted infectious particles falls below the detection limit. Thus, the observations initially made in undifferentiated Huh7 cells are confirmed in DMSO-differentiated Huh7 cells.

Because Reviewer #1 identified this as a major point, and given that this new finding is of interest to the community, these new data are now presented in Supplementary Fig. 3. The corresponding text describing these results has also been revised accordingly and integrated into the Results section as follows:

page 13, line 404-410 within the "Revised Article with Changes Highlighted" file:

"….To determine whether drugs targeting nicotinamide metabolism could alter viral replication in quiescent cells, Huh7 cells were differentiated for 7 days in the presence of 2% DMSO, and were then infected with HCV as previously described ([34]; Supplementary Fig. 3). In this model, treatment with 6-AN or FK866 reduced intracellular levels of viral RNA (Supplementary Fig.3A). Furthermore, and despite a limited effect on viral RNA secretion, the production of infectious particles collapsed (Supplementary Fig.3B and C). This inhibitory

effect was reversed by adding NAM to the culture medium, confirming the role of nicotinamide metabolism in HCV replication."

and page 14, line 447

"...A comparable inhibitory effect was also observed in Huh7 cells differentiated with DMSO prior to infection (Supplementary Fig. 3C)."

2. Do the inhibitors that target other parts of the NAD metabolic pathways also affect cell growth and ATP levels? If cell growth and ATP levels are reduced to a similar level by all inhibitors, the effect of 6-AN is rather specific. If not the reason for lower replication rates is likely due to the reduced cell growth and ATP levels unless the authors can demonstrate that that is not the case.

We found that Thio-NAD and phthalic acid have no detectable effect on either cell proliferation or intracellular ATP levels (Fig. R2). This is consistent with the predominant role of the NAD^+ salvage pathway in the liver (Xie et al. 2020) and the particularly high expression of NAMPT in hepatic tissue (Uhlén et al. 2015) .

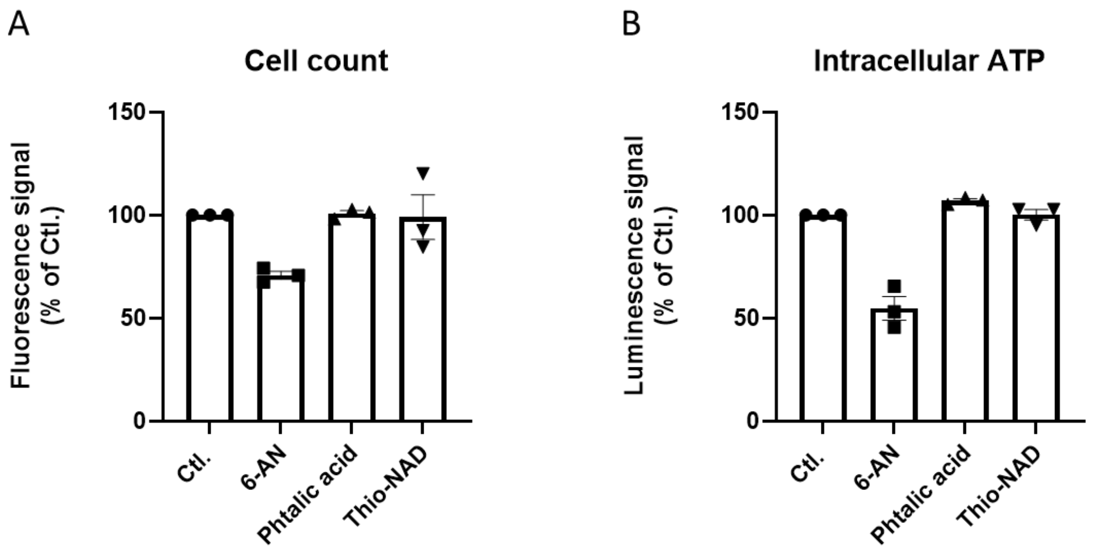

***Figure R2**: Huh7 cells were cultured for 72 h in the presence of 100 µM of 6-AN, phtalic acid or Thio-NAD. Intracellular ATP amounts were determined using CellTiter Glo assay (Promega) and cell proliferation was quantified after Hoechst staining of nuclei and measurement of fluorescence. Data correspond to the means ± SEM from 3 experiments*

We thank Reviewer #1 for this comment, which gave us the opportunity to clarify our manuscript. The decrease in ATP levels is correlated with the effects on the virus, but is not proportional to them. Indeed, an intermediate decrease in ATP (about 50% with 6-AN at 100 µM; Supp. Fig. 1A) is associated with an 80% inhibition of HCV replication (Fig. 1B) but more importantly, with a two-log decrease in the production of infectious particles (Fig. 3A). Quite similarly, a 50% reduction in both ATP levels and HCV replication in the presence of FK866 (Fig. 2) is associated with ≈1.5 log decrease in the production of infectious particles (Fig. 3D). Therefore, we agree that the decrease in viral replication is most likely a direct consequence of the metabolic slowdown due to the inhibition of the nicotinamide pathway,

which leads to a sharp reduction in the production of infectious particles. To clarify this point, the "Results" section entitled "HCV replication and DMV formation depend on NAD(H)" has been modified (Page 12, line 363 of the "Revised Article with Changes Highlighted" file):

The sentence " *Thus, 6-AN effectively restricts the replication of HCV in Huh7 cells, without affecting cellular viability.* " **was replaced by** "*…Thus, 6-AN effectively inhibits HCV replication in Huh7 cells, likely by limiting cellular metabolic activity and in particular ATP availability among other metabolites, while not affecting cellular membrane integrity. This shows that, without killing cells, 6-AN induces a metabolic slowdown limiting viral replication."*

This point is also more clearly addressed in the "Discussion" section of the manuscript. The following sentences have been added on Page 17, lines 563-571 within the "Revised Article with Changes Highlighted" file:

"Thus, decreasing the availability of NAD(H) broadly alters cellular metabolism, thereby reducing the capacity of cells to perform specific metabolic functions. Although this inhibition limits glycolysis and TCA cycle activity (Fig. 5), leading to decreased cell proliferation, it does not compromise cell viability or cellular integrity (Supplementary Fig.1). Inhibition of the NAD(H) also inhibits HCV replication, and this effect is particularly pronounced when considering the production of infectious particles and the specific infectivity of secreted virions (Figure 3). This suggests that multiple steps necessary to HCV replication cycle are impaired, leading to cumulative effects on the production of viral particles. We have also shown that a similar effect is observed in DMSO-differentiated cells (supplementary fig.3) ."

3. As 6-AN (and the other inhibitors) reduces HCV replication in replicon assays all downstream effects on virus assembly and release could be secondary. The authors should assess HCV release of cells that are chronically infected and then treated with the inhibitor for a short period of time. Ideally at cell density at the time of harvesting the supernatant should be similar.

As mentioned above, we agree with Reviewer #1 that the effects of 6-AN treatment on virus assembly and release are likely a consequence of a global metabolic slowdown. This is now clearly stated in the "Results" and "Discussion" sections (Page 12 and Page 17).

To evaluate the effect of NAD(H) metabolism inhibitors on chronically infected cells, Huh7 cells were infected at an MOI of 1 and cultured for 72 h to reach a plateau in viral RNA accumulation. The infected cells were then treated with 6-AN for 48 h, and the impact on viral replication and on the specific infectivity of particles produced under these conditions was assessed.

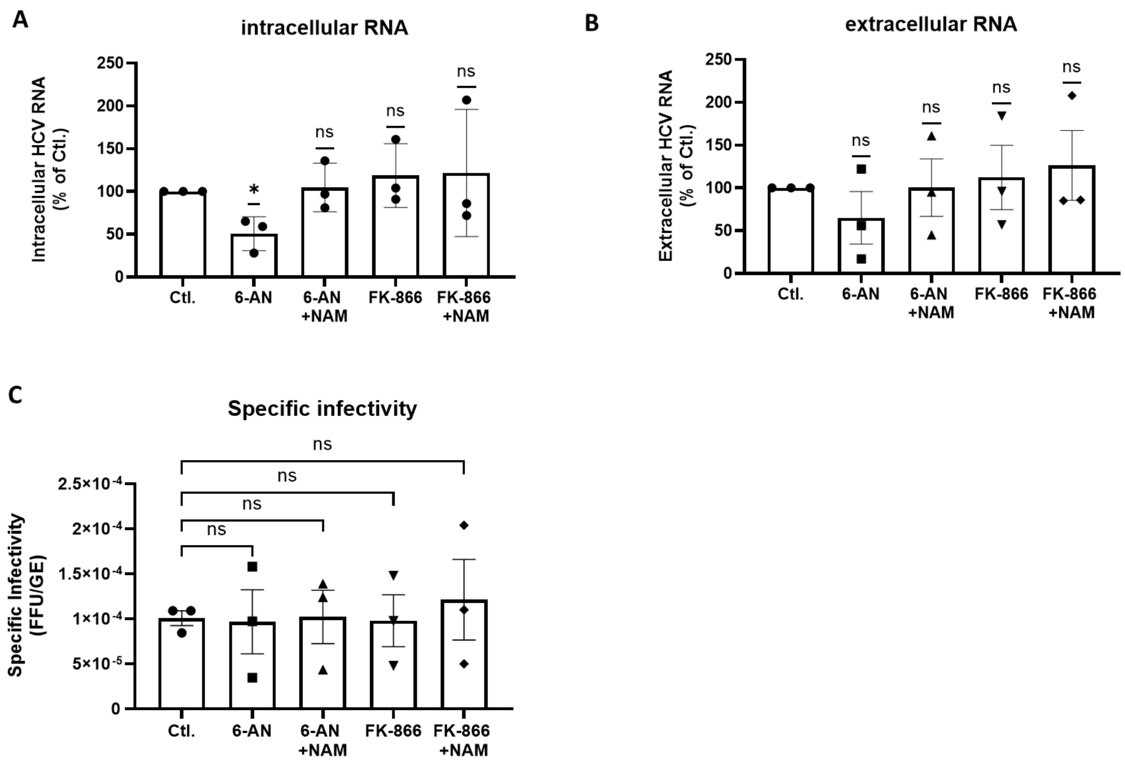

*Figure R3: Huh7 cells were seeded in 24-well plates, 24h before infection at a MOI of 1. Seventy-two hours post infection, culture medium was replaced and 6-AN (100 µM) or FK-866 (100 nM), supplemented or not with NAM (500 µM), were added to the cultures. After 48 h of treatment, intracellular (A) and extracellular (B) viral RNA were quantified by qPCR and specific infectivity of viral particles in supernatants was determined (C). Are presented means ± SEM (n=3). A and B, one sample t-test, *p<0.05,ns none significative. C, One-Way Anova with Sidak correction for multiple comparisons, ns none significative.*

Although an effect of 6-AN on intracellular RNA levels was observed, no effect of FK-866 was detected (Fig. R3A). Furthermore, we observed no effect of the inhibitors on either the quantity or the quality of secreted viral particles. These results indicate that nicotinamide metabolism plays a critical role during the establishment phase of HCV infection, while its contribution is limited once the infection is fully established. HCV is known to reprogram hepatocyte metabolism through virus-host protein-protein interactions to meet its energy and metabolite needs (Meyniel-Schicklin et al. 2012; Diaz et al. 2022; Perrin-Cocon et al. 2022) and see our answer to Question 5). Overall, our observations suggest that 6-AN or FK866 could interfere with these metabolic modulations associated with the early stages of infection, thus preventing the virus from establishing a fully productive infection. Conversely, once virus-induced metabolic reprogramming is fully established, the effect of these drugs is considerably reduced. This requires further investigation, and this will be carried out in future studies.

4. As explained above cancer cells have a different metabolic profile. Thus, the authors need to perform key experiments in primary human hepatocytes or hepatocytes differentiated from stem cells to confirm the relevance of NAD metabolism in a more physiological cell model.

We share with Reviewer #1 this methodological concern. We have carefully considered the integration of PHH or hepatocytes differentiated from stem cells into our experimental strategy. However, these models have significant technical and biological limitations,

including strong donor-to-donor variability, extremely limited availability for PHH, and reduced permissiveness to HCV, due to a very robust innate immune response. It was observed that following HCV infection, interferon-stimulated gene (ISG)-expression significantly increased in pluripotent stem cell (iPSC)-derived hepatocyte-like cells (iHLCs), whereas induction was almost absent in Huh7 (Schöbel et al. 2018). Although PHHs or iHLCs are of interest for the study of the interference of infection with innate immunity, a low susceptibility to infection was observed in both models. While iHLCs can support the full HCV life cycle, viral replication and RNA levels fall rapidly after infection, in contrast to Huh7 cells (Schöbel et al. 2018; Carpentier et al. 2020; Sakurai et al. 2017). In this model, as early as 24 hours after infection, a drop in viral replication is observed, which would not allow us to observe an inhibition by 6-AN. For PHHs, it is notoriously known that they are difficult to infect, with a low success rate and a weak secretion of infectious particles (Farquhar et McKeating 2008; Molina et al. 2008; Castet et al. 2002).

To answer Reviewer's comment, we attempted, without success, to infect PHH cells isolated from the margins of a human hepatocarcinoma resection (collaboration with the Hospice Civil de Lyon). A major limitation of this approach is that PHH are purified from cancer patients, and this pathological context, could profoundly affect the susceptibility of the hepatocytes to infection. We also had the opportunity to use HepaSH^{TM} cells (Biopredic International), which are human hepatocytes purified from the liver of immunocompromised mice engrafted with PHH (Uehara et al. 2023). This method allows the production of human hepatocytes that are more homogeneous than PHH cells obtained from cancer patients. In addition, the HepaSH cells display a similar level of drug-metabolizing enzyme activity and gene expression as the average PHHs ((Uehara et al. 2023)). Freshly purified cells were seeded in glycogen-coated well plates and culture cultured for 4 days in William-E medium (Gibco 12551032) containing 100µg/mL Penicillin/Streptomycin, 2mM L-Glutamine, 1% Non-Essential Amino Acids, Hydrocortisone, 5µg/mL Insulin, 2ng/mL EGF, 5% SVF, 2% DMSO and 10mM HEPES. Cells were infected at an MOI of 1, and intracellular HCV RNA levels were quantified 3 days post-infection (Fig. R4A). Secreted HCV RNA was also measured (Fig. R4B); although viral titers remained below the detection threshold, quantifiable levels of extracellular viral RNA were detected. Under these conditions, HCV infection was reduced upon 6-AN treatment, with a more pronounced effect observed on intracellular viral RNA than on secreted RNA. Supplementation with nicotinamide (NAM) restored extracellular viral RNA levels, while only partially recovering intracellular genome levels. Unfortunately, HepaSH cells are extremely expensive and are shipped fresh directly from Japan, and we were only able to perform this experiment once in the timeframe of the review process. Taken together, and despite the overall low level of infection in this experimental setting, these data support an inhibitory effect of 6-AN on HCV replication. If requested by the Reviewer or the Editor, we could include these results as a Supplementary Figure.

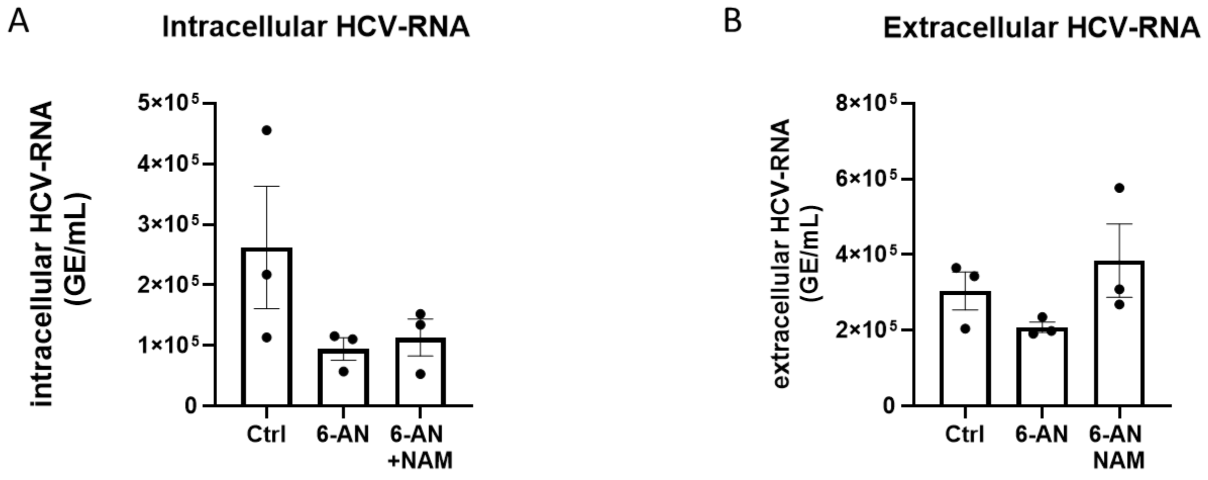

Figure R4: *HepaSH (donor FSSH003) were seeded in collagen-coated 24-well plates and cultured 4 days before infection at MOI=1. Twenty-four hours post-infection molecules or the solvent alone (Ctrl) were added at the final concentration of 100µM 6-AN ± 500µM NAM. Three days post infection, cells were harvested for RNA extraction and HCV genomes were quantified by qPCR. Are presented means ± SEM of 3 biological replicates proceeded in the same experiment.*

5. Does HCV infection itself affect the enzymes involved in NAD metabolism. Are gene or protein expression levels altered or is the enzymatic activity affected?

We analyzed publicly available transcriptomic data from HCV-infected hepatocytes obtained from the Gene Expression Omnibus (GEO) database. A time-course analysis on Huh7 cells infected with HCV (JFH-1 strain) over a period of 6 to 48 h.p.i. (GSE20948) shows that NAMPT transcripts are upregulated during infection (see figure R5 below). These data therefore indicate that infection enhances the expression of at least one of the limiting enzymes of NAD biosynthesis in the nicotinamide salvage pathway, further suggesting that this metabolic pathway is important for viral replication.

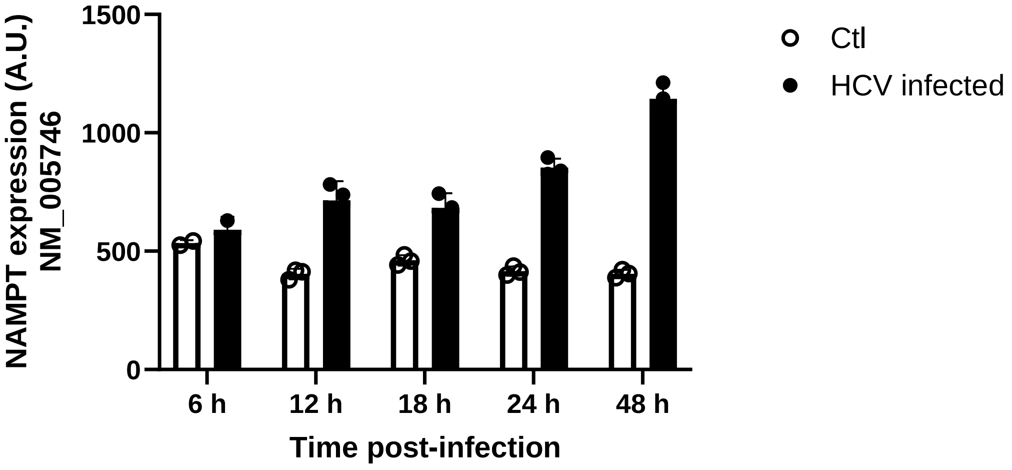

Figure R5*: Huh7 cells were seeded in 25-cm² culture flasks and infected in triplicate with HCV JFH-1 strain at an MOI of 3 or mock infected. At 6, 12, 18, 24 and 48 h post-infection, cellular RNA was extracted for expression analysis, where RNA was purified and hybridized onto Human U133 Plus 2.0 Affymetrix microarray chips for fluorescence data acquisition. The raw expression data from the microarray were processed using Array Studio software (Omicsoft) and extracted using the*

DataSet Browser interface on the NCBI website. Are represented data from n=3 of GSE20948 (ID: 81118025 for alignment with NAMPT (Gene ID:10135), NM_005746).

6. Figure 1 convincingly shows that HCV replication of the replicon cells is lower but is the RLU normalized to cell number or protein content of the replicon cells? Lower cell numbers could cause lower RLUs.

Data presented in Figure 1 are normalized to the luciferase activity measured in the absence of treatment. An 80% decrease in luciferase signal is observed when cells are treated with 100 µM of 6-AN (Fig. 1B), while cell count is only reduced by 30% as consequence of the inhibition of cell proliferation (Supplementary Figure 1B; Viability is not affected as shown in Supplementary Fig. 1C). Therefore, the dose-dependent effect of 6-AN on viral replication is more pronounced than its effect on cell proliferation (Supplementary Fig.1B). Therefore, the reduction in luciferase activity cannot be explained by the decrease in cell proliferation.

In response to the reviewer's suggestion, we normalized luciferase activity according to the number of cells present in each well at the time of luciferase activity measurement (Figure R6). Cell count was determined at the moment of luciferase assay by nuclear staining with Hoechst 33342 and fluorescence quantification, which served as an indicator of the number of cells. These normalized results confirm that the inhibitors of NAD metabolism strongly inhibit HCV viral replication by approximately 70% with 6-AN (Fig. R6A) and 66% with FK-866 (Fig. R6B) whereas NAM supplementation partially restore viral replication.

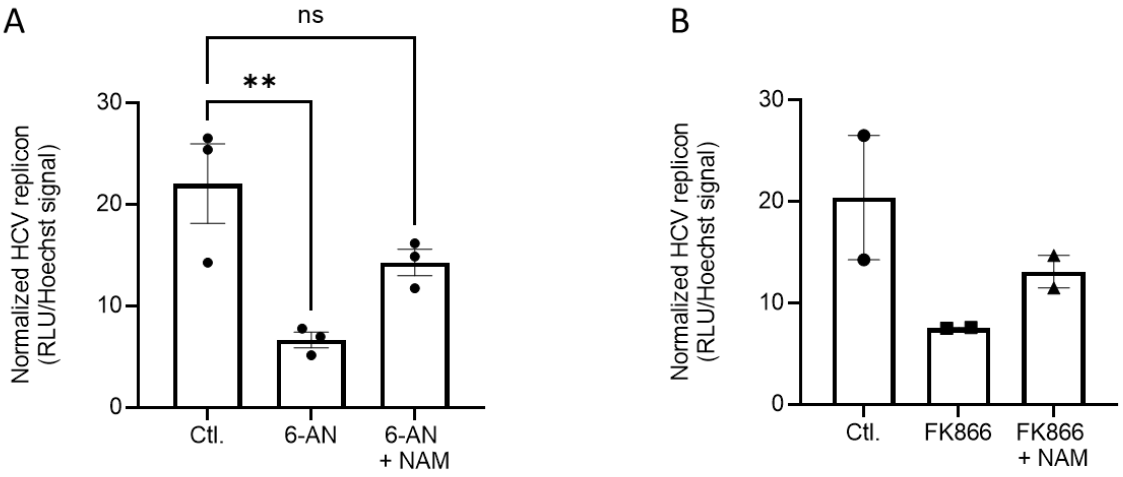

***Figure R6**: Huh7 cells were electroporated with the subgenomic luciferase replicon and seeded into white (luminescence) and black clear-bottom (Hoechst) 96-well plates. Treatments were added 4 h later, and cells were incubated for 72 h. Luciferase activity was measured after adding Bright-Glo™ reagent (Promega) and expressed as RLU. For cell quantification, Hoechst 33342 (40 µM) was added for 30 min at 37 °C, cells were washed with PBS, and fluorescence was measured with a Tristar 5 Multimode reader (Ex 350 nm/Em 461 nm). Are presented means ± SEM of 3 (6-AN) or 2 (FK866) independent experiments. One-way Anova **p<0.01, ns non significative.*

7. dsRNA staining can be used as a proxy for HCV RNA replication. Please perform IF with proper quantification of dsRNA foci (preferred) or quantify the amount membranous web area over several cells in EM images to assess if RNA replication complex formation in individual cells is less efficient.

To analyze the impact of the inhibition of nicotinamide metabolism on the number of replication complexes per cell, Huh7 cells were infected and then treated for 72 h with either 100 µM 6-AN or 6-AN supplemented with 500 µM NAM. Replication foci were

subsequently labeled using an anti-dsRNA antibody (clone J2; Scicons, Jena Bioscience RNT-SCI-10010200). Detection of replication complexes was achieved with an Alexa Fluor 488–conjugated secondary antibody, and cell nuclei were counterstained with Hoechst. Samples were analyzed by confocal microscopy (Figure R7).

The number and volume of replication foci per cell were quantified from Z-stack maximum intensity projections (Figure R7B). We observed that the percentage of cells with detectable replication complexes was significantly reduced after 6-AN treatment. This effect was reversed by the addition of NAM, which restored the proportion of cells exhibiting replication foci. Therefore viral spread in cultures was decreased when nicotinamide metabolism is inhibited..

In contrast, the number of replication foci per infected cell was not significantly affected by 6-AN, whereas their average volume was reduced in 6-AN–treated cells (Figure 7B). This indicates that although the number of replication foci per cell may remain unchanged, their size, and likely their activity, is reduced upon inhibition of nicotinamide metabolism. Such a reduction in replication complex size may explain the overall decrease in viral replication observed under these conditions.

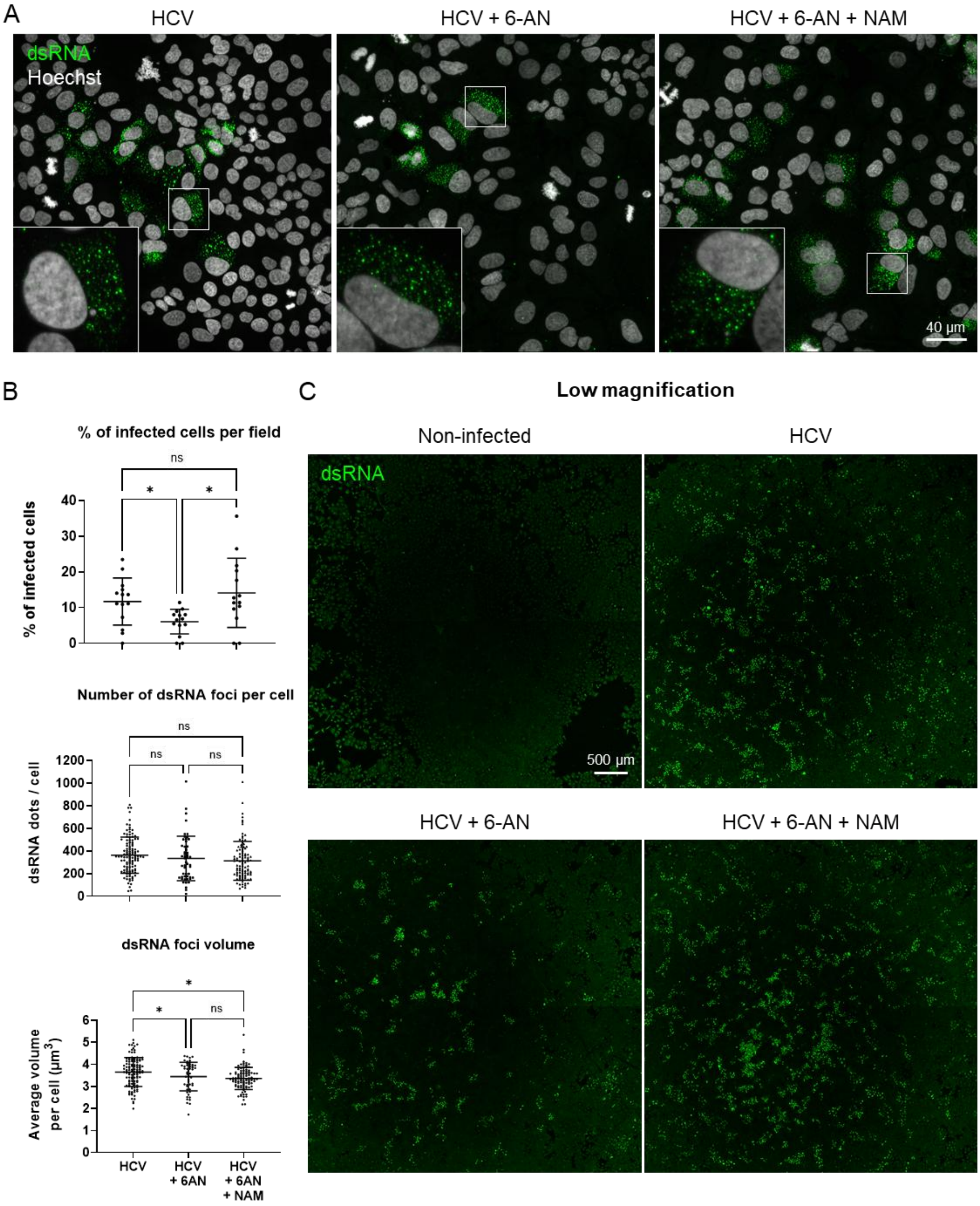

Figure R7: *Effect of 6-AN treatment on HCV dsRNA foci. Huh7 cells were infected and treated or not with 6-AN ± NAM, stained for dsRNA and imaged by confocal microscopy. (A) Representative Z-stack maximum intensity projections of cells stained for dsRNA. Closeups and whole field views (with stronger brightness and contrast adjustment to facilitate the visualization of foci-containing cells) are shown. (B) Corresponding quantification of the proportion of infected cells (dsRNA foci-containing cells), dsRNA foci abundance per cell and dsRNA foci volume. Data are presented as mean ± SD, and were analyzed using one-way ANOVA, *p<0.05, ns: not significative. (C) Mosaic microscopy images showing wide field views of the cell monolayers stained for dsRNA. Data are representative of 2 independent experiments.*

Thus we propose to include these novel analyses in the manuscript as Supplementary Figure 2. The numbering of the other supplementary figures has therefore been adjusted accordingly,

and the following paragraph has been added to the Results section “ HCV replication and DMV formation depend on NAD(H))”, page 12, line 370 in the “Revised Article with Changes Highlighted” file:

“*As a proxy of viral replication, we investigated the impact of 6-AN on viral replication complexes. We thus infected Huh7 cells with HCV (Jc1 strain) and treated cells for 72 h with either 100 µM 6-AN or 6-AN supplemented with 500 µM NAM. Replication foci were labeled using an anti-dsRNA antibody and visualized with an Alexa Fluor 488–conjugated secondary antibody, while nuclei were counterstained with Hoechst (Supplementary Fig.2). Confocal microscopy analysis revealed that the percentage of cells exhibiting detectable replication complexes was significantly reduced following 6-AN treatment, indicating impaired viral spread upon nicotinamide metabolism inhibition (Supplementary Fig.2B). This effect was reversed by NAM supplementation, which restored the proportion of cells displaying replication foci. Interestingly, the number of replication foci per infected cell was not markedly altered by 6-AN treatment. However, analysis of individual foci volumes showed that they were, on average, smaller in 6-AN–treated cells. These results suggest that while the number of replication complexes per cell remains largely unchanged, their size, and potentially their functional activity, is reduced when nicotinamide metabolism is inhibited. Such a reduction in replication complex size likely contributes to the overall decrease in viral replication observed under these conditions.”*

The following text describing the procedure of dsRNA staining and quantification has been added to the Materials and Methods section as follows, page 7, line 190 in the revised version of the manuscript:

“dsRNA foci staining and quantification. *Huh7 cells were seeded in ibidi µ-Slide 8-well chambers 24 h before infection (MOI 1) and treated or not with 100 µM 6-AN ± 500 µM NAM for 72 h. After removing the culture supernatant, cells were washed with PBS and fixed for 15 min at room temperature with 4% paraformaldehyde. They were permeabilized for 30 min at 4 °C using PBS + 0.2 M glycine + 1% Triton X-100, then blocked for 30 min at room temperature with PBS + 0.2 M glycine + 0.5% BSA. After a PBS + 0.2 M glycine wash, cells were incubated for 1 h at room temperature with anti-dsRNA antibody (1:100, clone J2, Jena Bioscience RNT-SCI-10010200) diluted in PBS + 0.2 M glycine. Cells were washed twice with PBS + 0.2 M glycine, incubated with Alexa Fluor 488-conjugated goat anti-mouse IgG (1:2000, Invitrogen A-11029) for 1 h at room temperature, washed again, stained for 5 min with 5 µM Hoechst in PBS, washed, and imaged on a Yokogawa HCS CQ1 confocal system. For dsRNA foci quantification, Z-stacks (11 µm total, 1.8 µm steps) were acquired in random fields (14 fields per condition from two independent slides) using a UPLSAPO40X2 objective (Olympus). Acquisition settings were 100% excitation and 750 ms exposure for the 488 nm channel, and 50% excitation and 500 ms exposure for the 405 nm channel. Images were analyzed in the CQ1 software using the “Dots in Cell body” template in slice mode to automatically segment dsRNA foci. Nuclei segmentation used the following steps: ThresholdToZero (400), Threshold (450), ErosionCircle (0.7 µm), DivideEachRegion, SizeFilter (450–20000 µm³), FillUp, ExcludeEdge. Cell bodies were segmented from green-channel autofluorescence using: MeanImage (5 µm mask), Threshold (150), DivideEachRegion, ExpandRegion3D (nucleus-guided), SizeFilter (20–10,000,000 µm³). dsRNA dots were segmented using: Gaussian filter (0.1 µm mask), Threshold (510), FindMaximumImage (remove size <0.3 µm), DivideEachRegion, ExpandRegion3D (Gaussian input; Threshold 450, 5 iterations), SizeFilter (0.1–1,000,000,000), and DilateRegion (0.1 µm). Low-magnification single-plane images were acquired using a UPLSAPO4X objective*

(Olympus) with the 488 nm laser (100% excitation, 2000 ms exposure), and stitched with the microscope software to generate mosaics covering nearly the entire well area."

8. Oil-red-O staining in isopropanol results in lipid droplet fusion. Please repeat the experiments using BODIPY or other state of the art lipid droplet stains to avoid artifacts in the quantification and characterization of lipid droplets.

We acknowledge that Oil Red O staining in the presence of isopropanol has been reported to occasionally induce fusion of lipid droplets in fixed samples (Fam et al. 2018), which may affect the accurate assessment of droplet volume and morphology. Although our analyses demonstrate a reduction in intracellular neutral lipid content, as indicated by lipid staining which is further corroborated by a specific enzymatic biochemical assay for triglycerides (see Fig. 6C), we carefully considered the reviewer's comment. Consequently, we have removed from the figure the quantification of mean lipid droplet volume, number, and sphericity (formerly Fig. 5E–G). We now focus on the total lipid droplet volume per cell (Fig. 6E) as a proxy for intracellular neutral lipid content, without attempting to infer lipid droplet structural properties. Together with TGs quantification (Fig. 6A), these results focusing on quantitative parameters that can be interpreted with confidence, indicate a decrease in lipid storage upon 6-AN treatment.

The text in the Results section, part "Inhibition of NAD(H) biosynthesis reduces cellular triglycerides level and secretion of apolipoproteins", has been modified as follows.

Page 16 Line 509 the sentence "*…Interestingly, average volume of LDs was reduced by 2-fold in cells treated with 6-AN consistent with intracellular quantification of TGs (Fig. 6A), whereas their number and sphericity remained unchanged (Fig. 6F and G), suggesting decreased in TG storage in cells without affecting LDs structure. ….*" **was replaced by** "*…Interestingly, the average LD volume per cell was reduced by 62% in 6-AN–treated cells (Fig. 6E), consistent with intracellular TG quantification using a specific biochemical enzymatic assay (Fig. 6A).*"

Reviewer #2: In general, it will be important for the authors to better emphasize that the effects of interfering with NAD metabolism is an indirect effect on HCV. In studies of host factors, we normally would disregard conditions where cellular metabolism and proliferation are significantly affected. I recognize that it in this case is a premise that NAD-dependent metabolism is what affects HCV infection. But this would have to more clearly stated and discussed, since the cells obviously are not in a good condition absent of NAD, which unsurprisingly affects viral replication.

Inhibition of NAD metabolism undeniably leads to a slowdown of central carbon metabolism of cells and this probably contributes to the inhibition of viral replication. This effect of the global decrease in cellular metabolism, which was addressed in the second paragraph of the Discussion, is now discussed more clearly in this section of the manuscript. The following sentences have been added page 17 lines 563-571 within the "Revised Article with Changes Highlighted" file:

"Thus, decreasing the availability of NAD(H) broadly alters cellular metabolism, thereby reducing the capacity of cells to perform specific metabolic functions. Although this inhibition

limits glycolysis and TCA cycle activity (Fig. 5), leading to decreased cell proliferation, it does not compromise cell viability or cellular integrity (Supplementary Fig.1). Inhibition of the NAD(H) also inhibits HCV replication, and this effect is particularly pronounced when considering the production of infectious particles and the specific infectivity of secreted virions (Figure 3). This suggests that multiple steps necessary to HCV replication cycle are impaired, leading to cumulative effects on the production of viral particles. We have also shown that a similar effect is observed in DMSO-differentiated cells (supplementary fig.3).”

On a related note, the authors state that “6-AN effectively restricts the replication of HCV in Huh7 cells without affecting cellular viability”. This needs to be rephrased in the light of 6-AN actually decreasing cell growth and metabolic activity. Although it may not disrupt membrane integrity of the cells already present, this decreased cellular metabolism clearly could (and does) explain the lowered HCV replication levels.

For clarity, we have replaced the term "cellular viability " with "cellular membrane integrity" in the sentence describing these results. To ensure an appropriate interpretation we have also rephrased the analysis at line 363, page 12 (“Revised Article with Changes Highlighted” file):

“…Thus, 6-AN effectively inhibits HCV replication in Huh7 cells, likely by limiting cellular metabolic activity and in particular ATP availability among other metabolites, while not affecting cellular membrane integrity. This shows that, without killing cells, 6-AN induces a metabolic slowdown limiting viral replication.”

Also, the 100nM of FK866 (or STF-118804) used has a significant impact on cellular metabolism and proliferation. Therefore, again, this cannot be described as a direct effect on HCV infection. Instead, the clearly affected metabolic state of the cell leads to an environment less conveying for HCV replication.

In agreement with this point, the text line 398-404, page 13 "*….As for 6-AN, FK866 and STF-118804 decreased intracellular ATP and inhibited cell proliferation, both reverted by medium complementation with NAM (Fig. 1C-F). This antiviral effect of these two inhibitors was reversed by the addition of NAM, thus demonstrating that NAMPT inhibition is indeed responsible for the antiviral activity of FK866 and STF-118804 (Fig. 2G and B)….*” **was replaced by** *“Similarly to 6-AN, FK866 and STF-118804 induced a marked reduction in intracellular ATP levels and a concomitant inhibition of cell proliferation, reflecting a global impairment of cellular metabolism. Both effects were fully reversed by supplementation of the culture medium with NAM (Fig. 1C–F). Importantly, the antiviral activity of FK866 and STF-118804 was also abrogated by the addition of NAM, demonstrating that inhibition of NAMPT is responsible for this phenotype. The resulting metabolic slowdown creates a cellular environment that is probably less permissive to viral replication.”*

Effects on virus production:
Whereas the effects on viral replication is clear, I am not convinced that 6-AN or FK866 treatment inhibits production of infectious particles as such.
Experiments on virus production (Fig. 3 and S3): Conclusions cannot be drawn as done here. It was already demonstrated that 6-AN and FK866 dramatically reduce viral replication (replicon data), including through inhibition of DVM formation. With a generally much attenuated replication level, it would obviously be expected that virus production is also highly affected. The results presented in Fig. 3 and S3 can therefore not be used to draw

conclusions on any effects of inhibition of the NAD pathway on viral assembly and release. Extracellular RNA may also be released e.g. from dying cells or through exocytosis. With this in mind, the statements on specific infectivity cannot be trusted.
Conclusions on this would instead require the density centrifugation of the viral particles released, and comparison of FFUs and viral RNA titers in the resulting fractions, as presented in Fig. 7. This data can then be used to make statements about reduced specific infectivity in the low-density fractions. However, not as to whether virus production levels as such are affected. In conclusion, it is OK to keep Fig. 3 and S3, however, only with the simple notion that, as expected from the reduced replication levels, virus production is also decreased.

We have taken this comment into account and have now revised the description of the results highlighted by the reviewer, which are now presented in Figure 3. In agreement with the reviewer's comment, we think that results presented in the Fig. 7 confirm that the specific infectivity of viral particles in density fractions is affected by treatments.

We thus modified as follows the text line 441, page 14 "…*Indeed, the ratio between infectious particles and secreted viral genomes (FFU/GE), shown in Figures 3C and 3F was markedly reduced for cells treated with 6-AN or FK866 respectively. As the FFU/GE ratio reflects the specific infectivity of the produced particles, this decrease suggests a reduced infective capacity of the virions, or at least a reduction in the number of infectious particles secreted, consistent with the inhibitory effect initially observed on viral replication (Fig. 1B-D) and DMV formation (Fig. 1E).*"

By extension, although perhaps obvious to analyze apolipoproteins, TGs and lipid droplets as done in Fig. 6, I would not find any associated differences to be causing the most relevant effects on HCV infection. Again, any impact on virus/LVP production would be less relevant if replication and DMV formation already is greatly impacted. This should be mentioned in the manuscript. Rather, given that lipid compositions are specifically regulated in DMVs as also discussed in this manuscript (see e.g. Bley 2020; DOI: 10.3390/ijms21082888), it would be of greater interest to investigate any role of the perturbations of lipid metabolism on DMV formation. This is something the authors should consider adding.

Indeed, it would be of interest to analyze the impact of lipid metabolism perturbation on the formation of DMVs. In the metabolomic analysis performed and presented in this manuscript (Fig. 4 and Supplementary Fig. 4), metabolites involved in phosphatidylethanolamine and phosphatidylcholine biosynthesis were significantly altered, as revealed by metabolite set enrichment analysis (MSEA). As an example, this analysis showed that choline and phosphocholine, both linked to phosphatidic acid (PA) synthesis, were increased in cells treated with 6-AN (Fig. R8 below). PA has recently been shown to be directly involved in the formation of HCV-induced DMVs (Tabata et al. 2021). The accumulation of two intermediates in its biosynthetic pathway may therefore suggest that PA production is altered in 6-AN–treated cells, which could contribute to the mechanisms underlying inhibition of DMV formation. To directly address this hypothesis, it would be necessary to induce DMV formation independently of viral infection and to perform a more detailed analysis of cellular membrane lipid composition, as well as to analyze the expression of enzymes involved in the synthesis of the relevant metabolic intermediates. Such analyses are beyond the scope of this study and need to be explored independently. This perspective is now explicitly mentioned in the Discussion at line 801, page 19, as stated in the following paragraph:

"….Given that the lipid composition of DMVs appears to be finely regulated (Bley et al. 2020), and that HCV has been shown to induce an increase in polyunsaturated fatty acids (PUFAs), such as arachidonic acid, docosahexaenoic acid, and eicosapentaenoic acid, as well as to modulate the elongation and desaturation activities of fatty acids incorporated into membrane phospholipids (Hofmann et al. 2018), it would be of interest to perform a detailed lipidomic analysis of cells treated with 6-AN. Specifically, it would be valuable to determine which molecular species within different phospholipid classes are selectively modulated upon NAD(H) metabolism inhibition. Likewise, analyzing the expression changes of enzymes involved in these biosynthetic pathways under these conditions would be informative. Such analyses should help to better define the broader impact of lipid metabolism modulation on DMV formation during viral infections, which will directly influence virus and LVP production."

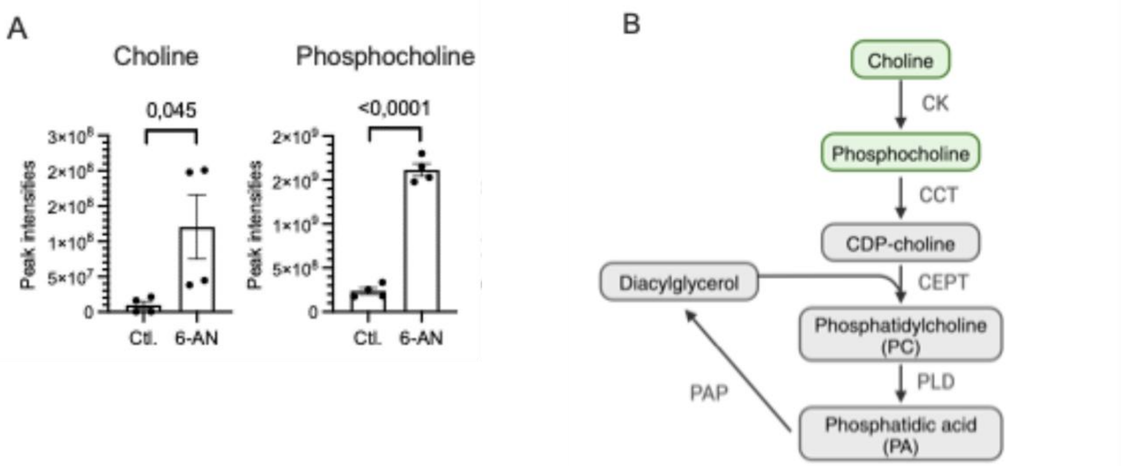

Figure R8 *: Huh7 cells were cultured for 72 h with or without 6-AN (100 µM) before metabolite extraction and analysis by LC-MS/MS. (A) Relative abundances of Choline and Phosphocholine. Means ± SEM obtained from 4 independent experiments are indicated with p-value for Student's t-test comparison. (B) Simplified diagram of the phosphatidylcholine biosynthesis pathway.*

Effect of 6-AN treatment on metabolism: Unlike what is stated in the Results section, the levels of NAD+ and NAM were not significantly reduced after 6-AN treatment (Fig. 4A). I suppose this is quite surprising given the theoretical expectation of 6-AN influencing NAD levels? Also, this questions the assumptions drawn from interpretation of Fig. 1-2, where it was concluded that 6-AN treatment affected HCV replication through decreasing NAD levels through the salvage pathway. To address this, the authors should perform intracellular quantification of NAD levels in the 6-AN and FK866 treated cells.

As requested by the reviewer, we quantified intracellular total NAD/NADH levels in cells treated with 6-AN or FK-866 with or without NAM supplementation, using the NAD/NADH-Glo™ Assay (Promega). This bioluminescent assay measures total NAD^+ and NADH with high sensitivity through an enzymatic cycling system in which NAD^+ is converted to NADH, enabling reductase-dependent generation of luciferin and subsequent luminescence proportional to total NAD content.

Intracellular NAD-dependent luminescence decreased by 24% in 6-AN–treated cells and by 97% in FK866-treated cells. In both cases, NAM supplementation restored NAD levels.

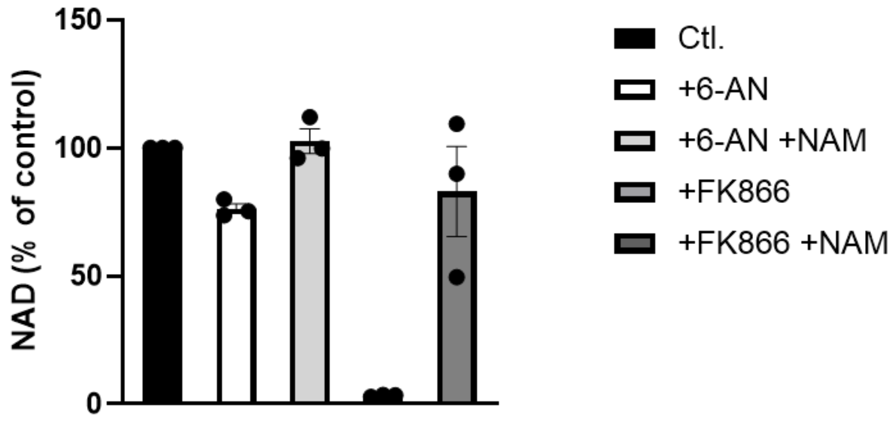

Figure R9: *Twenty-four hours after seeding, cells were treated for 72 h with 100 µM 6-AN or 100 nM FK866, with or without 500 µM NAM. After treatment, the culture medium was removed and the cells were washed with PBS. Total NAD⁺/ NADH was measured using the high sensitivity NAD/NADH-Glo™ Assay (Promega) as recommended by the manufacturer. For each treatment of cells, luminescence signal was measured and expressed as percentage of the untreated control. Are presented the mean ± SEM of three independent experiments.*

These results are consistent with the distinct mechanisms of action of the two compounds. FK-866 is a potent inhibitor of NAMPT, the rate-limiting enzyme of the nicotinamide salvage pathway, and therefore induces a profound depletion of intracellular NAD. In contrast, 6-AN acts as an antimetabolite that competes with nicotinamide and leads to the synthesis of 6-ANAD. Because it does not inhibit NAMPT directly, 6-AN results in only a modest reduction in NAD levels while still impairing NAD-dependent enzymes through 6-ANAD accumulation.

Part III – Minor Issues: Editorial and Data Presentation Modifications
Please use this section for editorial suggestions as well as relatively minor modifications of existing data that would enhance clarity.

Reviewer #1: 1. Most of the supplementary figures should be moved to the primary figures, e.g., Supp 1 D,E; Sup 2, Sup 3. That data could simply be merged.

Indicated supplementary figures have now been moved to the main figures. Previous Supp 1D,E and previous Supp 2 were added within Figure 1. Previous Supp 3 was added within Fig. 3. Figure panels numbering and corresponding mention in the text have been updated accordingly.

2. Why was Jc1-FLAG-E2 used for the experiments in Figure 7? The authors could just show Jc1 for consistency.

We performed 3 independent experiments with Jc1-FLAG-E2, while the experiment with Jc1 was carried out once. That's why we showed Jc1 in Supplementary Fig as a control.

Reviewer #2: That the "DMVs are the site of viral genome replication, capsid assembly, and envelopment" is perhaps attempting to state more than we know. The authors may want to

consult a newer review and qualify their statement based on current uncertainty of exactly how these processes are coupled and whether they indeed all involve the DMVs.

We thank the reviewer for this insight. We agree that, while DMVs are well established as the primary sites of HCV RNA replication, the precise spatial and temporal coupling of genome replication, capsid assembly, and LVP assembly is not fully resolved and remains an area of active investigation. We have revised the manuscript to better reflect the current understanding in the field. The revised text now states that DMVs serve as sites of viral genome replication and are closely associated with downstream steps of the viral life cycle, rather than asserting that all of these processes definitively occur within DMVs. These changes have been implemented in the manuscript

In the introduction at line 121.

"….*These DMVs serve as the sites of viral genome replication and are closely associated with downstream steps of virion assembly and lipidic envelopment.*"

In the discussion at line 578.

"…HCV-induced DMVs are well established as the primary sites of viral RNA replication and are closely associated with downstream steps of the viral life cycle, although the precise spatial organization and coupling of replication, assembly, and lipidic envelopment remain incompletely understood. Viral RNA replication occurs within DMVs enriched in the nonstructural proteins NS3, NS4B, NS5A, and NS5B, whereas virion assembly is thought to take place at endoplasmic reticulum (ER) membranes in close proximity to lipid droplets (LDs), involving the structural proteins core, E1, and E2 (Tabata et al. 2020). High-resolution live-cell imaging has revealed a selective recruitment of ER membranes around LDs, where structural proteins and components of the replication machinery colocalize (Lee et al. 2019). These ER membranes are decorated with DMVs, providing a potential topological framework to facilitate the spatial coordination of HCV genome replication and virion assembly. Nevertheless, how DMVs, assembly sites, and LDs are functionally coordinated remains unclear, particularly regarding the transport of viral components between compartments and the involvement of host lipid transfer enzymes (Tabata et al. 2020)."

I am not sure lipid droplets as such are required for HCV replication, as stated in the introduction? They clearly are for particle production.

To clarify our point, we have revised the final sentence of the Introduction as follows, line 138, page 5: "*We have notably established that targeting this host metabolic pathway prevents the formation of DMVs and reduces intracellular TG contained in lipid droplets, which are important for virion assembly and the production of LVPs.*"

Fig.7: In addition to the specific infectivity, it would be good to also depict the FFU titers as such across the collected densities. FFU titers were added to figure 7 as panel C.
