# Supplementary material for: Nicotinamide metabolism is essential for Hepatitis C Virus replication and the production of infectious Lipo-Viro-Particles
Source: PLoS Pathog. 2026 Apr 22;22(4):e1014165. doi: 10.1371/journal.ppat.1014165 (PMC13138740; doi:10.1371/journal.ppat.1014165)
Supplement: Attachment — Submitted filename: second response to reviewers.pdf [file ppat.1014165.s013.pdf]

PPATHOGENS-D-25-01682
Nicotinamide metabolism is essential for Hepatitis C Virus replication and the production of infectious Lipo-Viro-Particles PLOS Pathogens

Dear Editors,
Please find attached the revised version of our manuscript PPATHOGENS-D-25-01682, entitled "Nicotinamide metabolism is essential for Hepatitis C Virus replication and the production of infectious Lipo-Viro-Particles."
As recommended by you and the reviewers, we have included in the main text of the manuscript all of the data and figures that were generated during the reviewing process and initially provided only to the reviewers. All data generated during this study are now fully presented within the manuscript.
Below, we have provided a detailed, point-by-point response to the reviewers' comments. All concerns raised by both reviewers have been addressed.
All changes made to the manuscript are clearly indicated and detailed below. We sincerely thank the reviewers for their constructive comments, which have improved the quality of our work.
We hope that the revised manuscript is now suitable for publication.
Yours sincerely,
Dr. Olivier Diaz

Reviewer #1:

The authors addressed most issues. I recommend to include the new Supplemental Figure 2 in the Main Figures as part of Figure 1. In addition, Figures R3 and R4 should be added to the manuscript and discussed as these are important experiments that help to understand and validate their findings.

Supplementary figure 2 has been integrated into figure 1 (panels E, F and G) as bellow. References to this figure have been changed in the body of the text accordingly, and the caption of Figure 1 has been modified:

"***Figure 1:*** *HCV replication depends on NAD(H). (A) Schematic representation of NAD(H), NADP(H) biosynthesis pathways and targets of drugs used in the study. (B-D) Huh7 cells were electroporated with subgenomic replicon and cultured for 72 h with the indicated treatment. Then, Renilla luciferase activity was measured in cell homogenates and viral replication normalized to non-treated control (0 or Ctl.). Are presented means ± SEM. (B) Replicon-electroporated Huh7 cells were cultured in presence of increasing concentrations of 6-AN (0, 50, 100 and 500 µM), n=3, one-way ANOVA, Bonferroni-Sidák adjusted p-value for multiple comparison. (C) Cells were cultured with or without 100 µM of 6-AN and increasing doses of NAM, n=4, one-way ANOVA, Bonferroni-Sidák adjusted p-value for multiple comparison to 6-AN treatment. (D) Cells were cultured with or without 100 µM 6-AN and increasing doses of nicotinamide riboside (NR), n=3, one sample t-test, Bonferroni-Sidák adjusted p-value for multiple comparison to condition control. Are presented means ± SEM (n.s. non significative, *p<0.01, **p <0.01, ***p <0.001). (E) Huh7 cells were infected and treated or not with 6-AN ± NAM, stained for dsRNA and imaged by confocal microscopy. Representative Z-stack maximum intensity projections of cells stained for dsRNA. Closeups and whole fields of view (with stronger brightness and contrast adjustment to facilitate the visualization of foci-containing cells) are shown. (F) Corresponding quantifications of the proportion of infected cells (dsRNA foci-containing cells), dsRNA foci abundance per cell and dsRNA foci volume. Data are presented as mean ± SD, and were analyzed using one way ANOVA, *p<0.05, ns non-significative. (G) Mosaic microscopy images showing wide field of views of the cell monolayers stained for dsRNA. Data aggregate 2 independent experiments. (H) Huh7 cells*

were infected with HCV at MOI=1 and cultured with or without 100 µM 6-AN ± 500 µM NAM and prepared for observation by transmission electron microscopy. Red arrows indicate DMVs."

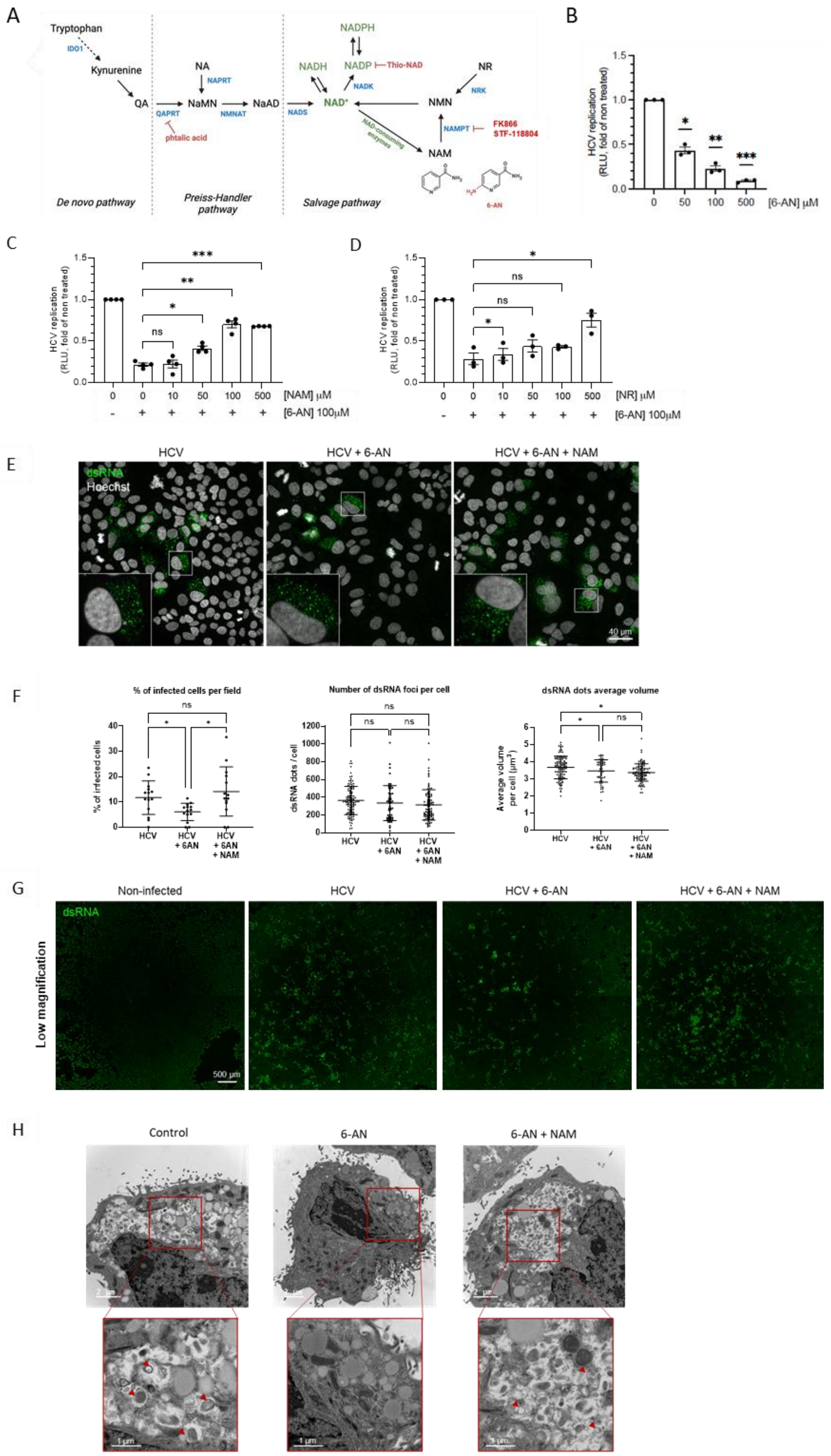

Figure R3, which was provided to reviewers during the first revision, is now integrated into the manuscript and constitutes Supplementary Fig.2 as below. The caption of this supplementary Fig.4 is « ***Supplementary Figure 4:*** *Huh7 cells were seeded 24h before infection at a MOI of 1. Seventy-two hours post infection, culture medium was replaced and 6-AN (100 µM) or FK-866 (100 nM), supplemented or not with NAM (500 µM), were added to the cultures. After 48 h of treatment, intracellular (A) and extracellular (B) viral RNA were quantified by qPCR and specific infectivity of viral particles in supernatants was determined (C). Are presented means ± SEM (n=3). A and B, one sample t-test, *p<0.05, ns non significative. C, One-Way Anova with Sidak correction for multiple comparisons, ns non significative."*

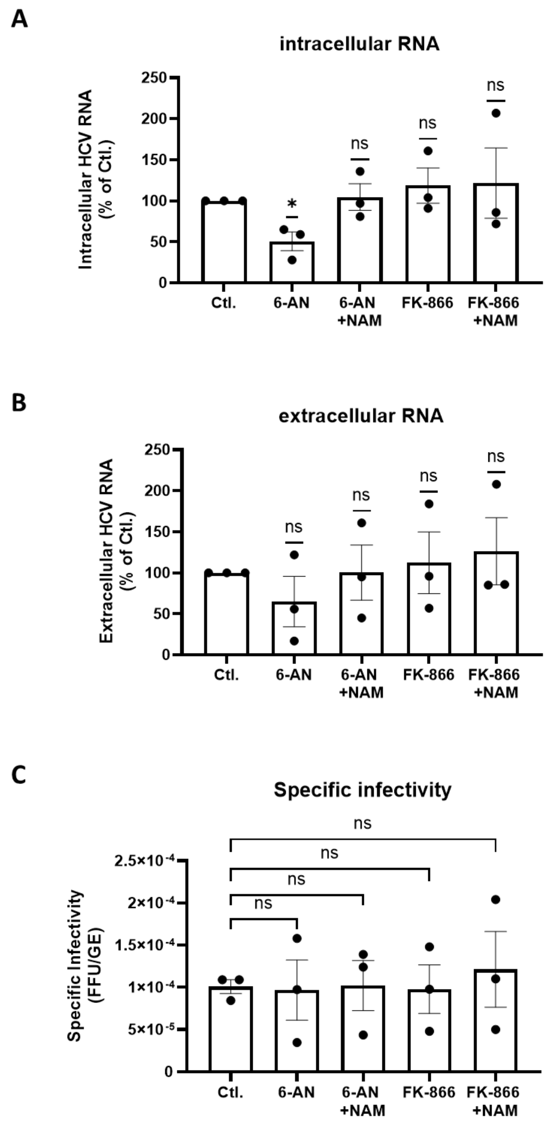

The analysis of data from Supplementary Figure 4 is presented in the body of the text, line 436 as follows: "…*We also sought to determine whether inhibition of nicotinamide metabolism could have an effect on infection once it was established, and not only during the initial phases of infection. To do this, we infected the cells at a MOI of 1, let the infection develop for 72 hours before treating the cultures with 6-AN or FK866 supplemented or not with NAM. Although an effect of 6-AN on intracellular RNA levels was observed, no effect of FK-866 was detected (Supplementary Fig. 4A). Furthermore, we observed no effect of the inhibitors on either the quantity or the quality of secreted*

viral particles (Supplementary Fig. 4B and C). These results indicate that nicotinamide metabolism plays a critical role during the establishment phase of HCV infection, while its contribution is limited once the infection is fully established."

These results are also discussed in the discussion part of the manuscript line 631: *"…Inhibition of the NAD(H) metabolism also inhibits HCV replication, and this effect is particularly pronounced when considering the initial phase of culture infection in contrast to latter stages when infection is established within the culture (supplementary Fig.4). Inhibitory effect on viral life cycle is also specifically important on the production of infectious particles and the specific infectivity of secreted virions (Figure 3). This suggests that multiple steps necessary to HCV replication cycle are impaired, leading to cumulative effects on the production of viral particles."*

Figure R4 is now integrated into the manuscript as Supplementary Fig.6 as follows:

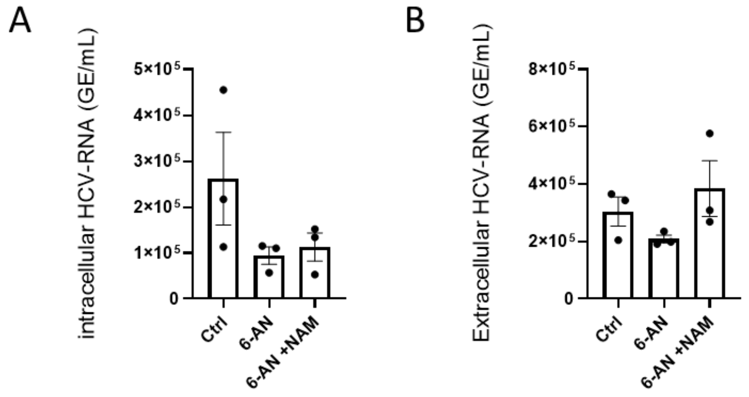

The caption of the supplementary Fig.6 is : *"**Supplementary Fig.6**: HepaSH cells were seeded in collagen-coated 24-well plates and cultured 4 days before infection at MOI=1. Twenty-four hours post-infection molecules or the solvent alone (Ctrl) were added at the final concentration of 100µM 6-AN ± 500µM NAM. Three days post infection, cells and culture supernatants were harvested for RNA extraction and HCV genomes were quantified by qPCR. Are presented means ± SEM of 3 biological replicates proceeded in the same experiment."*

The results of this supplementary Fig.6 are now described in the manuscript line 451: *"…We have also tested the effect of inhibition of NAD(H) metabolism in HepaSH cells, which are human hepatocytes purified from the liver of immunocompromised mice transplanted with primary human hepatocytes (PHH) (Uehara et al. 2023). Freshly purified cells were seeded in glycogen-coated well plates and cultured for 4 days prior to infection at a MOI of 1. Three days after infection, intracellular and secreted HCV RNA levels were quantified (Supplementary Fig.5); although viral titers remained below the detection threshold, quantifiable levels of extracellular viral RNA were detected. Under these conditions, HCV infection was reduced upon 6-AN treatment, with a more pronounced effect observed on intracellular viral RNA than on secreted RNA. Supplementation with NAM restored extracellular viral RNA levels, while only partially recovering intracellular genome levels. Taken together, and despite the overall low level of infection in this experimental setting, these data support an inhibitory effect of 6-AN on HCV replication in PHH."*

The culture method for HepaSH cells has been added in the material and method part as follows line 150: "***Human hepatocyte derived from chimeric mice (HepaSH) cultures.*** *Freshly purified cells were*

obtained from Biopredic International, seeded upon reception in glycogen-coated well plates as recommended by the supplier and cultured for 4 days in William-E medium (Gibco 12551032) containing 100µg/mL Penicillin/Streptomycin, 2mM L-Glutamine, 1% Non-Essential Amino Acids, Hydrocortisone, 5µg/mL Insulin, 2ng/mL EGF, 5% SVF, 2% DMSO and 10mM HEPES, before infection. "

Reviewer #2: In their point-by-point letter, the authors did a good faith effort in responding to the critique from the two reviewers. I notice also that both reviewers shared the major concern of differentiating between specific effects that interfering with the NAD pathways may have on HCV replication vs. general effects caused by attenuated cellular metabolism and proliferation, as well as the concern of interpreting effects on virus production in a setting of attenuated replication.

Part II – Major Issues: Key Experiments Required for Acceptance

Reviewer #1: The response to Q5 if the gene or protein expression or enzymatic activity are altered in infections was not sufficiently addressed. The authors could perform simple RT-qPCRs to analyze expression levels in their infection system and, importantly, present the data in the manuscript.

We quantified by RT-qPCR the expression of three key enzymes of nicotinamide metabolism, NAMPT, NAPRT and QPRT after 3 days of infection. NAMPT is the limiting enzyme of the nicotinamide salvage pathway and NAPRT and QPRT are required for the Preiss-Handler and the de novo NAD(H) synthesis pathway, respectively (Figure 1A). We did not observe any significant change in the expression of these three genes under our infection conditions (Supplementary Fig.2). Although some difference in NAMPT expression was reported in a transcriptomic study of Huh7 cells infected by HCV (Gene Expression Omnibus database GSE20948), these results are in accordance with public transcriptomic data from patients (GSE84346) . Using these data from Boldanova T et al. (PMID: 28360091), we compared the expression in liver biopsies of these enzymes between the 6 control individuals and the 20 HCV-infected patients who had not received any treatment. In this dataset, neither NAMPT, NAPRT nor QPRT were significantly increased during infection (Supplementary Fig.2D-E). Our results from RT-qPCR and public transcriptomic results from patients are now included in the Supplementary Fig.2 of the manuscript ; the analysis of these data has been incorporated into the manuscript line 405 as follows: "…*We first verified whether HCV infection modulated the expression of enzymes involved in this metabolic pathway. We quantified NAMPT, NAPRT, and QPRT by RT-qPCR in Huh7 cells after 3 days of infection. NAMPT is the rate-limiting enzyme of the nicotinamide salvage pathway, and NAPRT and QPRT are required for the Preiss-Handler and de novo NAD(H) synthesis pathways, respectively (Figure 1A). None of these enzymes was significantly modulated upon infection (Supplementary Fig. 2A-C). These results suggest that sensitivity to inhibitors of the nicotinamide metabolic pathway does not appear to be affected by viral infection. Furthermore, these observations are in agreement with transcriptomic data obtained from biopsies of patients infected or not by HCV (Boldanova T et al. (GSE84346)), and showing no significant difference in the expression of these three enzymes, in HCV-infected patients compared to non-infected patients (Supplementary Fig. 2D-F). Altogether, these observations suggest that sensitivity to inhibitors of the nicotinamide metabolic pathway does not appear to be affected by viral infection*".

The caption of Supplementary Fig.2 is :" ***Supplementary Fig.2***: *Huh7 cells were infected at MOI of 1. 72 h post-infection, total mRNA was extracted and NAMPT* ***(A)****, NAPRT* ***(B)*** *and QPRT* ***(C)*** *expression was determined by RT-qPCR using RPL13A as housekeeping gene. Gene inductions are presented as fold of control. Are presented means ± SEM of three independent experiments. Student t test, non-significative (ns).* ***(D-F)*** *Expression levels of NAMPT, NAPRT and QPRT in liver biopsies of control and HCV infected patients (Data extracted from* Boldanova T et al. study (PMID: 28360091; GSE84346)).

The description of RT-qPCR methods and specific primers used has been added to the material and methods section of the manuscript.

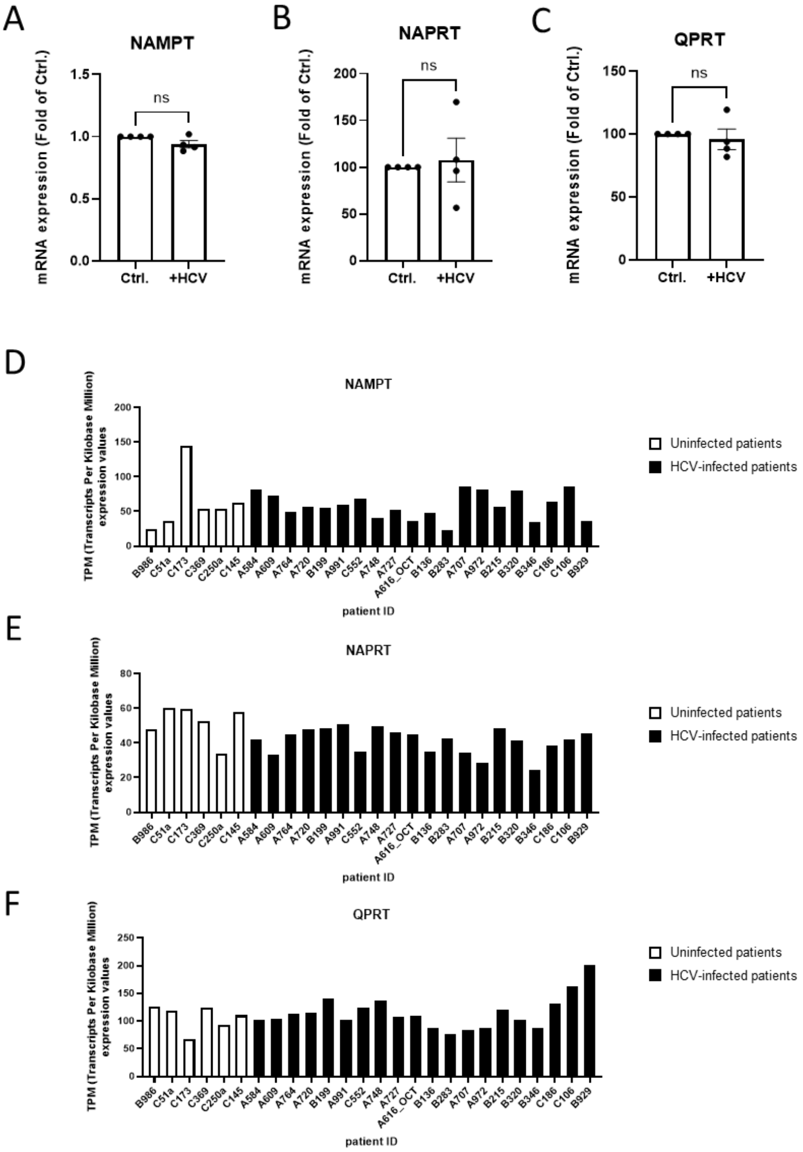

Reviewer #2: Curiously, despite the extra experiments performed to address reviewer questions, the authors decided against integrating many of these data sets into the revised manuscript. I would find it imperative that most of these data sets are added to the manuscript, such that not only the reviewers, but also the readers can make their own assessment with this data available.

As requested by the two reviewers, the data that had initially been presented into the answer to reviewers are now integrated into the manuscript (response to reviewer #1 above). In particular we now present in the manuscript, data obtained after infection of DMSO-differentiated cells (supplementary Fig.5) or HepaSH cells (supplementary Fig.6) as well as quantification of the expression of nicotinamide metabolism's enzymes (Supplementary Fig.2).

This would in particular include the data of Fig. R9 (Effect of 6-AN and FK866 on intracellular NAD levels). While FK866 substantially reduces NAD levels, the effect of 6-AN is surprisingly small,

challenging some of the NAD related conclusions made in the manuscript throughout. Although there is an effect, and the importance of this can be discussed, it would be important that the reader at least can assess this for themselves.

The quantification of NAD in cells after treatment with 6-AN or FK866 is now presented in the manuscript (Supplementary Fig.3 below) and is analyzed lines 418 of the manuscript as follows: "*…We quantified total NAD in cells treated with the antimetabolite 6-AN or the NAMPT inhibitor FK866. We used a bioluminescent assay measuring total NAD⁺ and NADH with high sensitivity through an enzymatic cycling system in which NAD⁺ is converted to NADH, enabling reductase-dependent generation of luciferin and subsequent luminescence proportional to total NAD cellular content. Intracellular NAD-dependent luminescence decreased by 24% in 6-AN–treated cells and by 97% in FK866-treated cells (**Supplementary Fig.3**). In both cases, NAM supplementation restored NAD levels. These results are consistent with the distinct mechanisms of action of the two compounds. FK-866 is a potent inhibitor of NAMPT, the rate-limiting enzyme of the nicotinamide salvage pathway which is the main provider of NAD in these cells, and therefore induces a profound depletion of intracellular NAD. In contrast, 6-AN acts as an antimetabolite that competes with nicotinamide and leads to the synthesis of 6-ANAD. Therefore, 6-AN results in only a modest reduction in NAD levels while still impairing NAD-dependent enzymes through 6-ANAD accumulation.”*

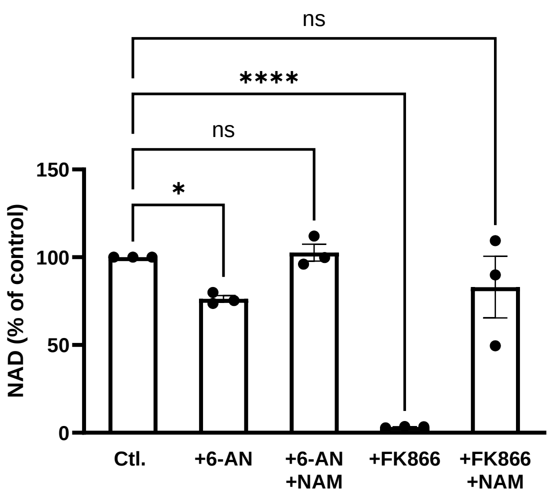

The caption of Supplementary Fig.3 is : “***Supplementary Fig.3**: Twenty-four hours after seeding, cells were treated for 72 h with 100 µM 6-AN or 100 nM FK866, with or without 500 µM NAM. After treatment, the culture medium was removed and the cells were washed with PBS. Total NAD⁺/NADH was measured using the high sensitivity NAD/NADH-Glo™ Assay. For each treatment of cells, luminescence signal was measured and expressed as percentage of the untreated control. Data are presented as the mean ± SEM of three independent experiments and were analyzed using one way ANOVA, *p<0.05, ****p<0.0001, ns none significative.*

In addition, I would find it important to include data from Fig. R3 (attempts to look specifically at effects on virus production), and Fig. R4 (Assessment of 6-AN in primary cells)

We agree with this and data are now in the manuscript as supplementary figures (see answer to reviewer#1)

Part III – Minor Issues: Editorial and Data Presentation Modifications

Reviewer #1: x-axis labelling is missing for Figures 1 C,D, 2 A,B.
Lane 395 should refer to Figure 2C-F.

Missing axis labelings are now present in final figures.

Reviewer #2: I feel less strongly about the data in Fig. R5 (Effect of infection on NAMPT abundance) and Fig. R2 (effect of other inhibitors on ATP abundance), however, since this data was generated, why not make it available?

Expression of NAMPT, NAPRT and QPRT was monitored by RT-qPCR and is now included in the manuscript in Supplementary Fig.2.

The effects of the different inhibitors on ATP abundance are now integrated into the manuscript in the Supplementary Fig.7 and introduced into the text as follows, line 473: « ... *Neither phthalic acid nor Thio-NAD had an effect on intracellular ATP or cell proliferation (Supplementary Fig.7).”*

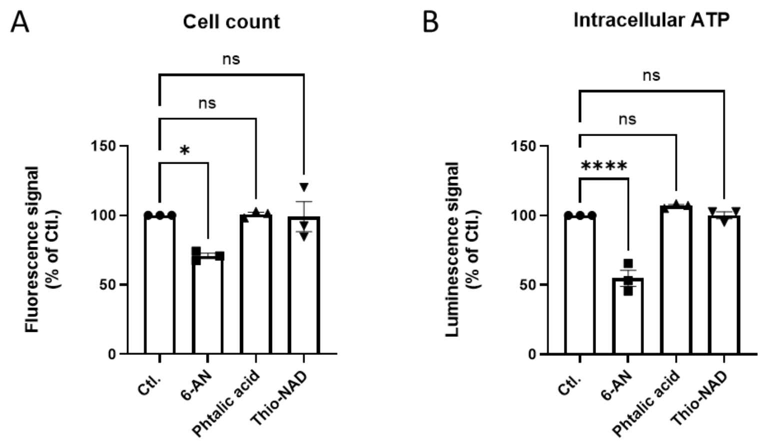

Caption of Supplementary Fig.7: “***Supplementary Fig.7:*** *Huh7 cells were cultured with or without 100 µM of 6-AN, Phtalic acid or Thio-NAD.* ***(A)*** *Cell proliferation was determined after Hoechst staining of nuclei and quantification of fluorescence.* ***(B)*** *Intracellular ATP amounts were determined using CellTiter Glo bioluminescent assay. Are presented means ± SEM, n=3, one-way ANOVA, Bonferroni adjusted p-value for multiple comparison. ns non significative, *p <0.05, ****p <0.0001.”*
